# Clinical and Genetic Analysis of Children with Kartagener Syndrome

**DOI:** 10.3390/cells8080900

**Published:** 2019-08-15

**Authors:** Rute Pereira, Telma Barbosa, Luís Gales, Elsa Oliveira, Rosário Santos, Jorge Oliveira, Mário Sousa

**Affiliations:** 1 Laboratory of Cell Biology, Department of Microscopy, Institute of Biomedical Sciences Abel Salazar (ICBAS), University of Porto (UP), Rua Jorge Viterbo Ferreira, 228, 4050-313 Porto, Portugal; 2 Multidisciplinary Unit for Biomedical Research (UMIB), ICBAS-UP, 4050-313 Porto, Portugal; 3 Department of Pediatrics, Maternal Child Center of the North (CMIN), University Hospital Center of Porto (CHUP), Largo da Maternidade de Júlio Dinis, 4050-651 Porto, Portugal; 4 Department of Molecular Biology, Institute of Biomedical Sciences Abel Salazar (ICBAS), University of Porto (UP), Rua Jorge Viterbo Ferreira, 228, 4050-313 Porto, Portugal; 5 Bioengineering and Synthetic Microbiology Group, Institute of Health Research and Innovation (IBMC/i3S), University of Porto (UP), Rua Alfredo Allen, 208, 4200-135 Porto, Portugal; 6 Center of Medical Genetics Dr. Jacinto de Magalhães (IGMJM), University Hospital Centre of Porto (CHUP), Praça Pedro Nunes 88, 4050-106 Porto, Portugal; 7 Center for Predictive and Preventive Genetics, Institute for Molecular and Cell Biology (IBMC), Institute of Health Research and Innovation (i3S)-UP, Rua Júlio Amaral de Carvalho 45, 4200-135 Porto, Portugal; 8 UniGene- IBMC/i3S, UP, Rua Alfredo Allen, 208, 4200-135 Porto, Portugal

**Keywords:** primary ciliary dyskinesia, *situs inversus*, whole-exome sequencing, *CCDC40*, *DNAH5*, *DNAH7*

## Abstract

Primary ciliary dyskinesia (PCD) is a rare autosomal recessive disorder characterized by dysfunction of motile cilia causing ineffective mucus clearance and organ laterality defects. In this study, two unrelated Portuguese children with strong PCD suspicion underwent extensive clinical and genetic assessments by whole-exome sequencing (WES), as well as ultrastructural analysis of cilia by transmission electron microscopy (TEM) to identify their genetic etiology. These analyses confirmed the diagnostic of Kartagener syndrome (KS) (PCD with *situs inversus*). Patient-1 showed a predominance of the absence of the inner dynein arms with two disease-causing variants in the *CCDC40* gene. Patient-2 showed the absence of both dynein arms and WES disclosed two novel high impact variants in the *DNAH5* gene and two missense variants in the *DNAH7* gene, all possibly deleterious. Moreover, in Patient-2, functional data revealed a reduction of gene expression and protein mislocalization in both genes’ products. Our work calls the researcher’s attention to the complexity of the PCD and to the possibility of gene interactions modelling the PCD phenotype. Further, it is demonstrated that even for well-known PCD genes, novel pathogenic variants could have importance for a PCD/KS diagnosis, reinforcing the difficulty of providing genetic counselling and prenatal diagnosis to families.

## 1. Introduction

Motile cilia are responsible for mucociliary clearance [1], cerebrospinal-fluid local flow [2], sperm motility [3], and oocyte transport [4]. Ciliary motility is mainly due to the 9 + 2 ciliary axoneme that is a complex structure, composed by nine peripheral microtubule doublets (A, B) that are linked by nexin bridges and present dynein arms (DAs) (ODA: outer and IDA: inner), which are linked to a central microtubule pair by radial spokes (RS). The central pair, surrounded by a fibrillar sheath and connected by a central bridge is collectively known as the central pair complex (CPC) [5].

Genetic defects in ciliary proteins or proteins that are required for ciliary function lead to improper cilia function, resulting in a wide range of diseases collectively known as ciliopathies. Primary ciliary dyskinesia (PCD, MIM #244400) is a ciliopathy caused by dysfunction of motile cilia and is a rare autosomal recessive disease [6]. The abnormal motile cilia function results in several airways complications, such as chronic bronchitis and bronchiectasis [7]. *Situs inversus* is also frequent and the combination with PCD is designated as Kartagener syndrome (KS). Moreover, subfertility or infertility are often described, in both males and females [8,9,10]. PCD is; thus, a highly heterogeneous disease and patients can show a multiplicity of clinical features and disease severities. Further, there is no standard diagnostic for PCD and thus the final diagnosis requires a combination of several tests, such as the ultrastructural analysis of the ciliary axoneme, high-speed video microscopy analysis, or quantitative determination of the variation in ciliary beat axis and the ciliary deviation, and genetic screening [7]. Genetic screening requires high-throughput techniques, such as whole-exome sequencing (WES), which is a faster and more cost-effective approach [11,12].

We report a comprehensive clinical, ultrastructural, and genetic characterization by WES of two children with clinical features compatible with KS. WES identified heterozygous variants in *CCDC40*, *DNAH5*, and *DNAH7* genes. This was followed by additional mRNA and protein expression studies, which suggested that those could account for the observed PCD phenotypes. Besides the elaboration of a genetic diagnosis for patients, results enabled the discovery of six new genetic variants in those genes and suggest a role of gene interactions between genes *DNAH5* and *DNAH7*.

## 2. Materials and Methods

### 2.1. Ethical Issues

All ethical guidelines were followed, with written informed consent having been obtained from the parents beforehand. No further ethical or institutional approvals were needed as patient samples and databases are included in the regular assessment of the patients. This work did not involve human or animal experiments. The provisions of the Declaration of Helsinki as revised in Tokyo 2004 do not apply to this work. Regarding the laboratory work, the Joint Ethics Committee CHUP/ICBAS approval number is 2019/CE/P017(266/CETI/ICBAS).

### 2.2. Patients

Two unrelated children were followed at the Maternal Child Centre of North/Hospital and University Centre of Porto (CMIN/CHUP), due to upper respiratory complaints and *situs inversus* totalis. Both parents are healthy and non-consanguineous, and both pregnancies and deliveries occurred without problems. The present age of the parents of Patient-1 is 45 years (mother) and 52 years (father). The present age of the parents of Patient-2 is 32 years (mother) and 36 years (father). Children did not present neonatal transient tachypnea, neonatal pneumonia (meconium aspiration), and signs of surfactant protein deficiency (respiratory insufficiency at birth) or interstitial lung disease. Respiratory function tests were not performed due to age. The alpha-1-antitrypsin assay, the immunological study, and the atopy test were normal. Cystic fibrosis was also excluded.

#### 2.2.1. Patient-1

This female child, currently 7 years of age (birthday date (BD): 28.07.2011), was born at 39 weeks of gestation by vaginal delivery. At birth, she presented an Apgar score of 8/10, a birth weight of 3190 g (z-score −0.09), a length of 50 cm (z-score 0.46), and a head circumference measure of 35 cm. She was referred for consultation at 16 months of age due to acute bronchiolitis of difficult resolution, followed by recurrent respiratory infections, some requiring hospitalization due to exacerbation. In the first moments of life, cardiac sounds were observed on the right side, with chest X-ray evidencing an inversion of the cardiac silhouette and gastric chamber on the right, raising the suspicion of a *situs inversus totalis*. Later, she developed poor weight evolution, recurrent acute otitis media (AOM), mucopurulent rhinorrhea, and productive daily cough, with no signs of asthma. During development, the child did not acquire chest deformities or digital clubbing, discarding chronic respiratory insufficiency development, and the pulmonary auscultation remained normal. Currently, she weighs 20.4 Kg (z-score −0.61), has a height of 120 cm (z-score −0.15), and a head of circumference of 50 cm (z-score 0). She is under daily respiratory kinesiotherapy and sporadic cycles of antibiotic therapy.

#### 2.2.2. Patient-2

This male child, currently 7 years of age (BD: 05.11.2011), was born at 39.5 weeks of gestation by vaginal delivery (with a suction cup). At birth, he presented an Apgar score of 10/10, a birth weight of 2958 g, a (z-score −0.84), a length of 52 cm (z-score 1.12), and a head circumference measure of 34.5 cm (z-score 0). He was hospitalized with respiratory distress syndrome (up to day 13) and right opacification on the chest X-ray compatible with right pneumonia, without any microbiological agent being identified. He presented clinical improvement after an antibiotic therapy cycle. During hospitalization, cardiac sounds were found on the right during auscultation. Chest X-ray confirmed the inversion of cardiac laterality with the presence on the right of the gastric chamber, raising the suspicion of *situs inversus totalis*. During the neonatal period (up to day 28), virus screenings in secretions were negative. Up to about 2.5 months of age, control chest X-ray did not evidence opacities compatible with pneumonia, having developed nasal obstruction, productive cough, purulent nasal secretions, without wheezing, fever or AOM. From 1 year of age, he developed sporadic and self-limiting crises of a productive cough and wheezing, without fever, AOM, or mucopurulent rhinorrhea. During evolution, the child did not develop chest deformities or digital clubbing, which is compatible with the absence of chronic respiratory insufficiency, and the pulmonary auscultation remained normal. Currently, he weighs 27.4 Kg (z-score 1.25), has a height of 121 cm (z-score −0.14), and a head circumference of 52.5 cm (z-core 1.25). He is under daily respiratory kinesiotherapy.

### 2.3. Sample Collection

Genomic DNA was extracted from peripheral blood leukocytes using the salting-out protocol [13]. Respiratory epithelial cells were obtained by nasal brushing, using a cytology soft sterile brush (Endobrush, Biogyn SNC, Mirandola, Italy), in both nostrils [14]. Children were premedicated with oral paracetamol (15 mg/kg/dose) before nasal brushing. All individuals were continuously monitored for vital signs, pain complaints, and/or signs of bleeding.

### 2.4. Sample Processing for Transmission Electron Microscopy

Nasal samples were fixed as described elsewhere [14]. Briefly, samples were fixed with 2.5% glutaraldehyde (Sigma-Aldrich, St. Louis, MO, USA) in 0.1 M cacodylate buffer (Merck, Darmstadt, Germany), pH 7.2, 2 h, room temperature (RT), and post-fixed with 2% osmium tetroxide (Merck), dehydrated in graded ethanol series (VWR, Monroeville, PA, USA), and treated with 1% tannic acid (Merck) in 100% ethanol prior to embedding in epoxy resin (Epon, Sigma-Aldrich). Suitable areas of ciliated cells were selected in semithin sections (1 µm) and stained with methylene blue-Azure II (Merck). Semithin and ultrathin sections were cut on an LKB-ultramicrotome (Leica Microsystems, Wetzlar, Germany) using diamond knives (Diatome, Hatfield, PA, USA). Ultrathin sections were retrieved on copper grids (Taab, Berks, England). After contrasting with aqueous uranyl acetate (BDH, Poole, England) and lead citrate (Merck), they were observed in a JEOL 100CXII transmission electron microscope (JEOL, Tokyo, Japan), operated at 60 kV.

### 2.5. Cilia Morphological Evaluation and Orientation

The ultrastructure of cilia axonemes was evaluated based on the presence of a systematic defect in any of the axonemal structures [5].

Variation in ciliary beat axis and the ciliary deviation was evaluated in a minimum of 100 transverse sections examined after printing. In printed images, a perpendicular line to the central microtubules was drawn. A reference line was then chosen based on the main orientation of the lines drawn. The angle of each line to the reference line was calculated and subtracted to the mean. These differences are near zero. The standard deviation (SD) of these differences corresponds to the variation in ciliary beat axis and ciliary deviation [15]. Measurements were conducted always by the same observer.

### 2.6. Whole-Exome Sequencing

Exomes were sequenced using the AmpliSeq strategy on an Ion Proton next-generation sequencing (NGS) platform (Life Technologies, Thermo Fisher Scientific, Carlsbad,, CA, USA) and variant calling was performed as described [16]. The complete quality parameters and variant metrics of the exome runs are summarized in Table S1. All variants were listed in a Variant Call Format (VCF) file that was annotated and filtered using the Ion Reporter Software version 5.2 (http://ionreporter.lifetechnologies.com/) and VarAFT 2.10 (http://varaft.eu) (VarAFT, Aix Marseille University, Marseille, France). Alamut Visual v2.10 software (Interactive Biosoftware, Rouen, France) assisted variant interpretation. To analyze variants obtained, an autosomal recessive disease model was used. We firstly filtered-out the more deeply intronic-placed variants, synonymous substitutions, and variants with allele frequency above 1% in human population databases, assuming that those variants are improbable to be deleterious [17]. Further, we excluded genes with only one heterozygous variant and selected the variants based on the following gene ontology [18] keywords: cilia/flagellum; cilia assembly/morphology/motility; axoneme; dynein; intraflagellar transport; left-right axis and symmetry; and development. Further, *HYDIN axonemal central pair apparatus protein* (*HYDIN)* gene variants were also excluded, due to the high false discovery rate in mutation calling of this gene [19].

All the suspected variants were manually checked on the Binary Alignment Map (BAM) file through GenomeBrowse version 2.0.2 (Golden Helix, Bozeman, MT, USA). Sanger sequencing was applied to validate the candidate variants, as previously reported [11], with the primers listed in Table S2.

For the variants found in *DNAH7*, we performed a structural analysis using in silico tools. For that, a combination of cytoplasmic dynein structural models was used: human cytoplasmic dynein-1 (PDB ID:5NUG) [20] and dynein-2 (PDB ID:4RH7) [21], *Dictyostelium discoideum* cytoplasmic dynein 1 (PDB ID:3VKH) [22] and *Saccharomyces cervisiae* cytoplasmic dynein 1 (PDB ID:4AKI) [23]. Clustal Omega (https://www.ebi.ac.uk/Tools/msa/clustalo/) and ESPript (http://espript.ibcp.fr/ESPript/ESPript/) were used for multiple sequence alignment and to obtain secondary structure information from aligned sequences, respectively. The figures were rendered with protein data bank (PDB) data and the PyMol program. Additionally, homozygosity mapping (HM), using the web-based tool HomozygosityMapper (http://www.homozygositymapper.org/) [24], was performed.

### 2.7. Gene Expression Analysis

Total RNA from nasal cell suspensions was extracted using the NZY Total RNA Isolation kit (NZYTech, Lisbon, Portugal), according to manufacturer’s instructions and including the optional step of “DNase treatment”. The concentration and purity of RNA samples were determined on a Nanodrop spectrophotometer ND-1000 (Version 3.3; Life Technologies), with the cut-off A260/A280 ratio between 1.8 to 2.1. The RNA to complementary DNA (cDNA) conversion was done with the High Capacity cDNA Reverse Transcription kit (Applied Biosystems, Foster City, CA, USA), according to manufacturer’s instructions.

Real-time quantitative PCR (qPCR) was performed to evaluate mRNA expression of *DNAH5* (NM_001369.2) and *DNAH7* (NM_018897.3) genes in nasal cells of Patient-2 and from his parents. *ECM7* (NM_020154.3) was used as a housekeeping gene to normalize gene expression levels [25]. The primers used in qPCR reactions are listed in Table S2. qPCR was performed in a Bio-rad CFX96 (Bio-Rad, Hercules, CA, USA) and amplifications were prepared with the NZY qPCR Green (NZYTech). Three technical replicates were performed in each PCR assay. Fold variation of gene expression levels was calculated following a mathematical model using the formula 2 − ΔΔCt [26]. The statistical significance was determined using the non-parametric statistical test Mann–Whitney, with alpha < 0.05, Two-tailed *p*-value. Mann–Whitney is the nonparametric counterpart of the t-test. The results from the Mann–Whitney test were further confirmed using Dunn’s multiple comparisons test. Tests were performed in the GraphPad Prism (version 6.01, GraphPad Software, San Diego, CA, USA). Samples from the Patient-2 and his parents were compared to samples from individuals without PCD/KS (designated as controls). 

### 2.8. Immunofluorescence Analysis

The immunofluorescence (IF) analysis of nasal epithelial cells was performed as described previously [27]. Briefly, cell suspensions were spread onto glass slides (STARFROST, Knittel-Glass, Bielefeld, Germany), air dried, and stored at −80 °C until use. Cells were fixed with 4% paraformaldehyde (20 min, RT) (Merck) in PBS pH 7.4 (Panreac, Barcelona, Spain), permeabilized in 0.2% Triton X-100 (Sigma) (15 min, RT), blocked with 5% non-fat milk (60 min, RT) (Nestlé, Vevey, Switzerland), and incubated overnight at 4 °C, with antibodies rabbit anti-DNAH5 and anti-DNAH7 (Biorbyt, Cambridge, United Kingdom) and mouse anti-acetylated α-tubulin (Santa Cruz Biotechnology). For each experiment, a negative control was included by omitting the primary antibody. Anti-rabbit IgG Alexa Fluor 488 (Invitrogen, Carlsbad, CA, USA) and anti-mouse IgG Texas Red (Santa Cruz Biotechnology, Dallas, TX, USA) were used as secondary antibodies. Cells were counterstained with Vectashield mounting medium containing 4′,6-diamidino-2-phenylindole (DAPI: Vector Laboratories, CA, USA). Results were observed in an epifluorescence microscope (Eclipse E400; Nikon, Tokyo, Japan).

The level of cellular fluorescence from fluorescence microscopy images was determined using the ImageJ software (version 1.52 h, NIH, Bethesda, MD USA). Using the measurements obtained from the ImageJ software, we calculated the corrected total cell fluorescence (CTCF) (CTCF = integrated density – (area of selected cell × mean fluorescence of background readings)). The statistical analysis and the graphic were performed in the GraphPad Prism. Statistical significance determined using the Mann–Whitney test, with alpha < 0.05. ** = *p* value < 0.01; *** = *p* value < 0.001. The results from the Mann–Whitney test were further confirmed using Dunn’s multiple comparisons test.

## 3. Results

### 3.1. Echocardiogram and Thoracoabdominal CT Scan

**Figure 1:**
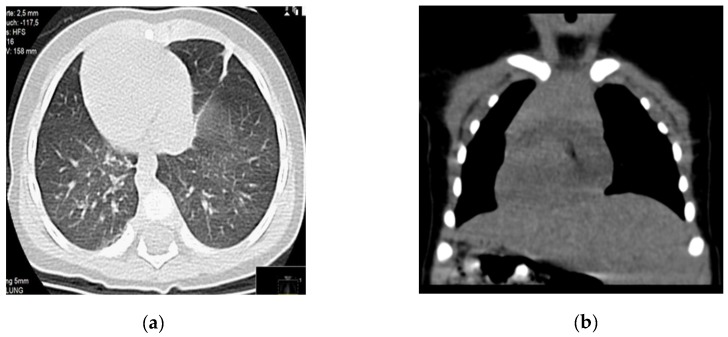
Patient-1 CT scan without contrast. (**a**) Dextrocardia and normal lung parenchyma. (**b**) Dextrocardia and left-sided liver.

In both children, *situs inversus totalis* was confirmed after an echocardiogram and a thoracoabdominal CT scan, which showed a complete inversion of the heart and lungs, the presence of liver on the left and of stomach and spleen on the right (Figure 1). No structural intracardiac anomalies were detected in the echocardiogram.

### 3.2. Transmission Electron Microscopy of Cilia

In Patient-1, the ultrastructural analysis revealed that most of the axonemes showed a mixed absence of dynein arms, with a predominance of the IDA absence in about 62.5% of the cases observed at high resolution. In all cases, nexin bridges were absent and radial spokes presented an undefined structure. At a lower resolution, an inspection of 356 axonemes revealed 32.3% of cases with normal localization of the central pair, 44.7% of the cases showed displacement of the central pair, and 23% of the cases revealed a disrupted axonemal structure (Figure S1A,B). As high-speed phase-contrast video imaging was not accessible at our facilities, we calculated the variation in ciliary beat axis and ciliary deviation, which was proved to be valuable in the determination of cilia function [15]. Evaluation of 192 axonemes revealed an SD of 43.60 (36.4–50.7), which is compatible with a PCD severity in cilia axis movement [15].

The ultrastructural analysis of cilia axonemes in Patient-2 showed an absence of both DAs and nexin bridges in all sections, with the presence of RS and CPC (Figure S1A,C). Evaluation of 228 axonemes revealed a variation in ciliary beat axis and ciliary deviation SD of 18.4, which is compatible with a bronchiectasis severity in cilia axis movement, a milder PCD feature [15].

### 3.3. Whole-Exome Sequencing

#### Whole-Exome Sequencing Variant Interpretation and Bioinformatic Analysis

In Patient-1, we found two compound heterozygous variants in the *CCDC40* gene: One substitution in a splice site, NG_029761.1(NM_017950.3):c.1989 + 1G > A, that has a paternal origin; and one frameshift variant, NM_017950.3:c.2824_2825insCTGT;p.(Arg942Thrfs*57), inherited from her mother (Figure S2A; Table 1). The first variant, although already listed in the Exome Aggregation Consortium (ExAC) database with a frequency of 0.0008%, was here firstly reported in the ClinVar database (SCV000537859.1). From the best of our knowledge, there are no publications associating this variant to PCD. This variant was predicted to affect the wild-type donor splice site by all splice site prediction algorithms included in the Alamut software (Figure S2B). The variant NM_017950.3:c.2824_2825insCTGT was listed in the ExAC database with a frequency of 0.0042% and in ClinVar, but like the previous one, no publications have reported this variant before. Although a similar variant was published, that leads to an in-frame insertion, NM_017950.3:c.2824_2825insTGT;p.(Arg942delinsMetTrp) [28]. In contrast, the variant that we have identified leads to a frameshift insertion and thus is predicted to affect the open reading frame.

**Figure 2:**
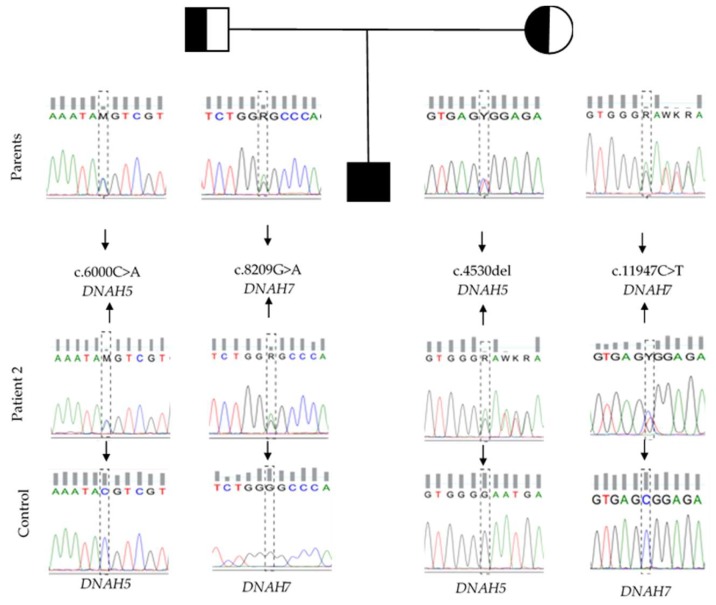
Pedigree and sequencing electropherograms of the variants in genes *DNAH5* and *DNAH7* identified in this study.

In Patient-2, four compound heterozygous variants were found: Two in the *DNAH5* gene and two in the *DNAH7* gene (Figure 2; Table 1). In the *DNAH5* gene, we have found one frameshift variant caused by a single base deletion at the coding position 4530 (NM_001369.2: c.4530del; p.(Asn1511Metfs*6)) that was inherited from his mother; and one a nonsense variant received from his father, which was caused by a change from cytosine to adenine at the coding position 6000 (NM_001369.2: c.6000C > A; p.(Tyr2000*)). Both SNV were here firstly detected and reported on the ClinVar database (submission numbers: SCV000579338.1 and SCV000537861.1, respectively). Regarding the *DNAH7* gene, two missense variants were found. One is a very rare (0.0008%) missense variant, inherited from his father, at position 8209 (NM_018897.3: c.8209G > A; p.(Gly2737Ser)), which leads to a change from a hydrophobic glycine to a polar serine on amino acid 2737. The other, transmitted from his mother, was found within the cDNA position 11947, involving the replacement of the positively charged arginine to the hydrophobic tryptophan (NM_018897.3: c.11947C > T; p.(Arg3983Trp)).

**Figure 3:**
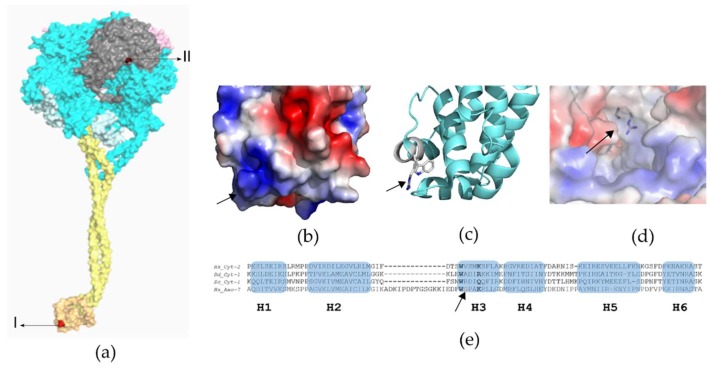
Structural analysis of *DNAH7* variants. (**a**) General dynein motor domain structure: N-terminal linker (pink), six AAA domains in ring arrangement (blue), stalk (yellow) containing the MTBS (orange), C-terminal (grey). Mutations (red) p.Gly2737Ser (I) and p.Arg3983trp (II). Crystallographic model PDB ID 4RH7: Crystallographic structure of human cytoplasmic dynein 2 motor domain. (**b**,**c**) Crystal structure of the MTBS of *D. discoideum* cytoplasmic dynein-1 (PDB ID: 3VKH): The p.Gly2737Ser mutation is in H3 (arrows). H3 is highly polar and assumes an important role in the interaction with the microtubule: electrostatic surface shown in (**b**) and H3 key residues in (**c**). (**d**) Close-view of the C-terminal of the crystallographic structure of human cytoplasmic dynein-2 motor domain (PDB ID 4RH7). The R side chain is disordered in the 4RH7 model as it is exposed to solvent. The putative position (arrow) of the p.Arg3983trp mutation highlights the superficial position of the residue. (**e**) Multiple sequence alignments: Hs_Cyt-2-human cytoplasmic dynein-2 (PDB ID: 4RH7), Dd_Cyt-1-*D. discoideum* cytoplasmic dynein-1 (PDB ID: 3VKG), Sc_Cyt-1-*S. cerevisiae* cytoplasmic dynein-1 (PDB ID: 4AKI), and Hs_Axo-7- human axonemal dynein-7. The secondary structure of Hs_Cyt-2 is indicated (alpha-helices H1 to H6). The arrow is pointing to the amino acid (glycine, G) that is mutated in Patient-2.

Variants found in *CCDC40* and *DNAH5* genes have a high potential to be disease-causing and allow to confidently predict the consequences at the protein level. Regarding the missense variants in the *DNAH7* gene, the direct consequence of an amino acid substitution is less clear. Therefore, to gain a better understanding of the molecular alterations introduced by the variants in the *DNAH7* gene, we structurally localized the mutant residues. Because the atomic structure of axonemal dyneins has not been solved, the location of the mutations was predicted using multiple sequences and structural alignments from a combination of cytoplasmic dynein structural models, whose structures were already solved, specifically: human cytoplasmic dynein-1 [20] and dynein-2 [21], *D. discoideum* cytoplasmic dynein-1 [22], and *S. cerevisiae* cytoplasmic dynein-1 [23]. The conformational variations revealed by these sequences are thought to be related to the different nucleotide states due to the power stroke, an essential mechanism to dynein movement [21]. Therefore, conformational changes are visible depending on if structures were obtained in pre-power stroke or in post-power stroke state [29]. This analysis revealed that the mutation inherited from his father, p.(Gly2737Ser), is located in an ATP-sensitive microtubule-binding site (MTBS). Concerning the mutation inherited from his mother, p.(Arg3983Trp) is placed in the C-terminal region (Figure 3).

Both variants found in the *DNAH7* gene were predicted to be disease-causing according to several prediction tools (Table S3), and both affected amino acids are well conserved throughout evolution (Figure S3). Additionally, homozygosity mapping (HM) was performed in both patients to determine the existence of genome long homozygous stretches. In our patients, longer stretches of homozygous single nucleotide variants were not identified, corroborating the compound heterozygosity of both cases (Figure S4) [30,31].

### 3.4. DNAH5 and DNAH7 mRNA Relative Expression

**Figure 4:**
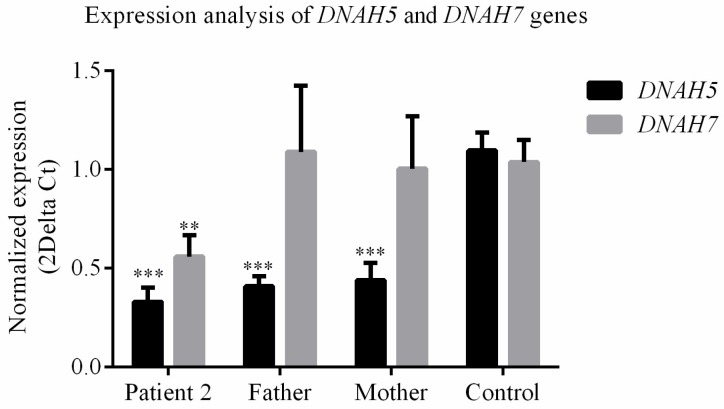
RT-PCR analysis of *DNAH5* and *DNAH7* mRNA expression levels in epithelial respiratory cells from Patient-2 and his parents, compared to healthy donors (a fluorescent dye used: SYBR Green). *ECM7* was used as the reference gene. Statistical significance determined using the Mann–Whitney test, with alpha < 0.05. ** = *p* value < 0.01; *** = *p* value < 0.001.

To explore the molecular consequences of the genomic variants identified in *DNAH5* and *DNAH7* genes from Patient-2, we analysed mRNA expression in nasal epithelial cells from this patient and his parents compared to controls. The *DNAH5* mRNA relative expression of Patient-2 decreased by 0.22-fold of the control amount (Figure 4).Likewise, in the carrier’s father and mother, we observed a decrease by 0.27-fold and 0.28-fold of the control amount, respectively (Figure 4). This suggests that those variants are harmful enough to, per se, affect gene expression. The *DNAH7* mRNA relative expression of Patient-2 decreased by 0.53-fold of the control amount (Figure 4). Carrier parents did not show significant differences in *DNAH7* mRNA relative expression in comparison to controls. 

### 3.5. DNAH5 and DNAH7 Immunofluorescence Analysis

To further assess the functional significance of the detected variants in the *DNAH5* gene, as well from both missense variants in the *DNAH7* gene, we performed IF analysis in respiratory cells of Patient-2 and his carrier parents.

**Figure 5:**
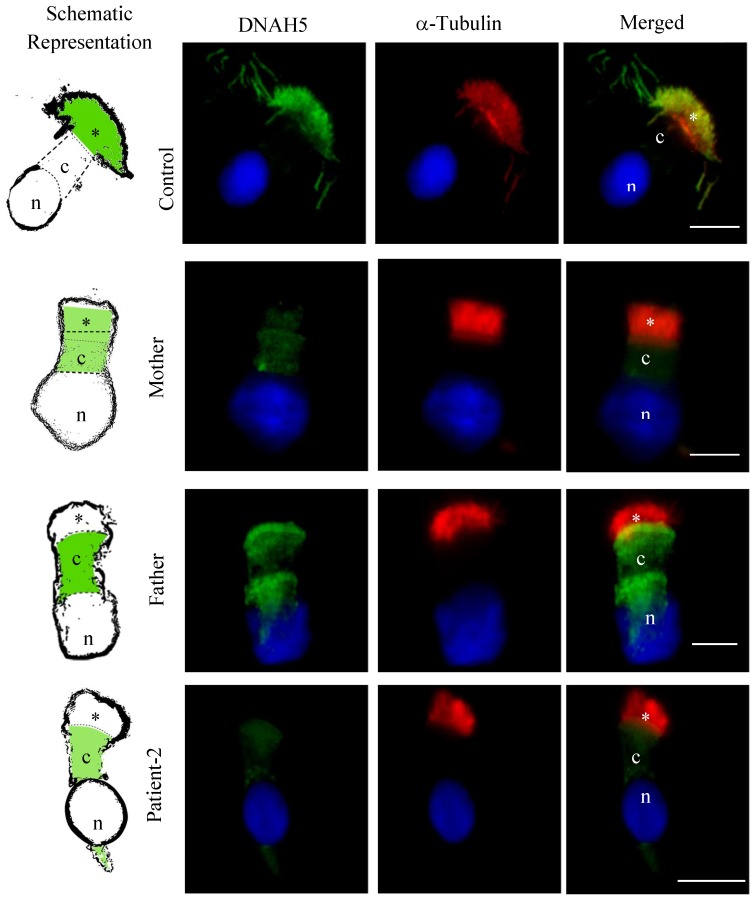
Expression and localization of specific DNAH5 protein by immunofluorescence in respiratory epithelial cells from healthy volunteers (Control), both parents of Patient-2 (Mother and Father) and Patient-2. Staining with anti-DNAH5 antibodies (green) and with antibodies against axoneme-specific acetylated α-tubulin (red). Nuclei stained with DAPI (blue). Schematic representation at left to better localize the staining (DNAH5 in intense or faint green). * = cilia; c = cytoplasm; n = nuclei. Scale bars = 10 µm.

**Figure 6:**
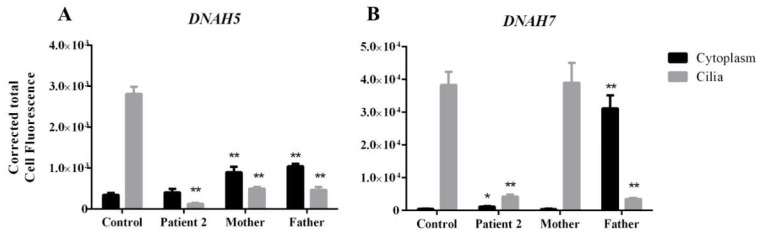
Corrected total cell fluorescence from immunofluorescence images obtained after immunofluorescence experiences with DNAH5 (**A**) and DNAH7 (**B**) antibodies, and calculated from the measurements obtained from the ImageJ software. Statistical significance determined using the Mann–Whitney test, with alpha < 0.05. * = *p* value< 0.05 ** = *p* value < 0.01.

In cells from healthy volunteers, DNAH5 intense staining was observed only in cilia axonemes. In Patient-2, we saw a significant reduction in staining of the DNAH5 in cilia axonemes (0.005-fold decrease of the control amount; *p* = 0.0043) and noted weak cytoplasmic staining. In the same way, in both parents, a significant reduction in staining of the DNAH5 in cilia axonemes (0.18-fold decrease in father and 0.17-fold decrease in mother of the control amount; *p* = 0.0043) was noted. Moreover, in both Patient-2 parents, an increased in staining within the cytoplasm (2.58-fold increase in father and a 3-fold increase in mother of the control amount; *p* = 0.043) was also observed (Figure 5 and Figure 6). 

**Figure 7:**
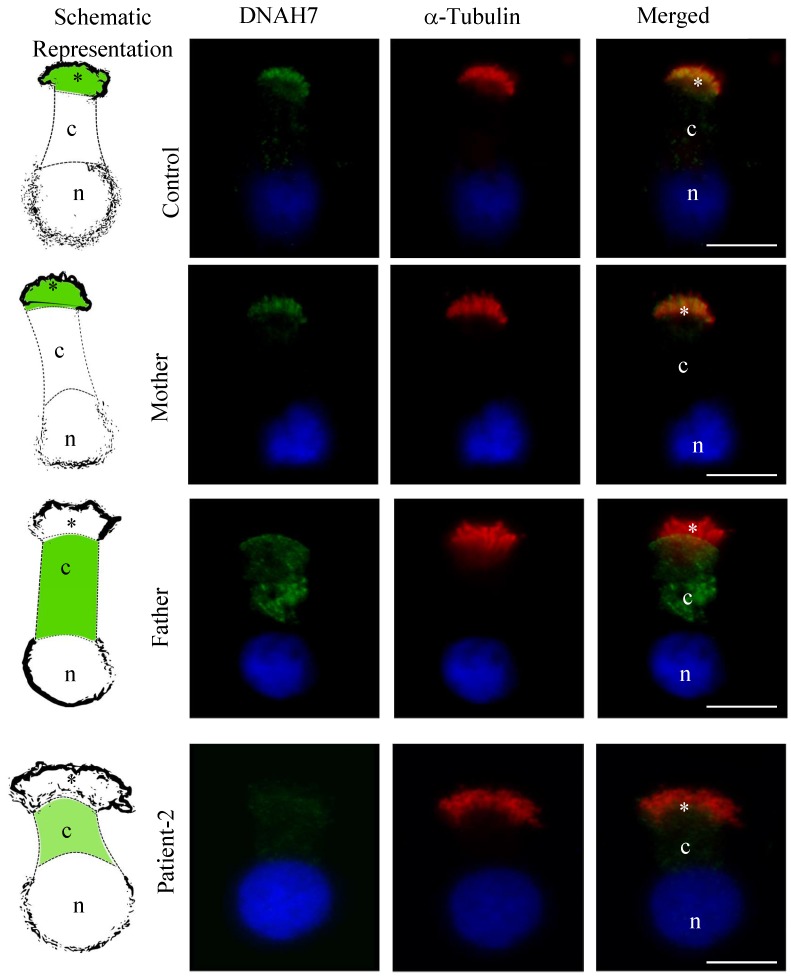
Expression and localization of specific DNAH7 protein by immunofluorescence in respiratory epithelial cells from healthy volunteers (Control), both parents of Patient-2 (Mother and Father) and Patient-2. Staining with anti-DNAH7 antibodies (green) and with antibodies against axoneme-specific acetylated α-tubulin (red). Nuclei stained with DAPI (blue). Schematic representation at left to better localize the staining (DNAH7 in intense or faint green). * = cilia; c = cytoplasm; n = nuclei. Scale bars = 10 µm.

Regarding DNAH7, in cells from healthy volunteers, staining was observed throughout the cilia axonemes. In Patient-2, we noted a significant reduction in staining within cilia axonemes (0.11-fold decrease; *p* = 0.0043), and faint cytoplasmic staining (a slight increase of 1.6-fold of the control amount; *p* = 0.0173). In the mother, staining was detected in the cilia axonemes, with no differences to control individuals. In the father, a significant reduction in staining of the DNAH7 in cilia axonemes was observed (0.1-fold decrease of the control amount; *p* = 0.009), and a significant increase of the staining within the cytoplasm (a 50-fold increase of the control amount; *p* = 0.0043) (Figure 6 and Figure 7).

## 4. Discussion

In the present report, two children with PCD and *situs inversus totalis* were characterized, whose definitive diagnosis was given after combined ultrastructural analysis and genetic screening.

In Patient-1, ultrastructural analysis of cilia axonemes revealed a heterogeneous pattern of cilia axoneme ultrastructural anomalies, and two disease-causing compound heterozygous variants in the *CCDC40* gene were described. Previous studies in animal models suggested that *CCDC40* plays an important role in cilia function and left-right axis specification [28]. Further, was proposed that *CCDC40* requires the formation of a complex with *CCDC39* to function properly [32]. The CCDC39-CCDC40 complex was proposed to have an important involvement in the docking of each IDA, RS, and nexin bridges at its correct position [32]. Consquently, genetic anomalies in *CCDC40* or *CCDC39* were described to impair the *CCDC39–CCDC40* complex, which leads to multiple ultrastructural ciliary anomalies [28,32].

The variant c.1989 + 1G > A is predicted to affect the donor splice site. The donor splice site is a key regulator of splicing and includes a highly conserved GU sequence. This variant implies a change in the GU residue (GU → AU), and likely impairs the binding of the U1 small nuclear ribonucleoprotein. This could potentially cause exon skipping [33] or activate a cryptic splice site (i.e., a sequence identical to that of the donor splice site but that is not regularly used). In both probable scenarios, an abnormal RNA [33] was formed.

The other variant, c.2824_2825insCTGT, lead to a complete shift of the reading frame and putatively to a non-functional protein. Unfortunately, during the time of this study, we were unable to obtain a new nasal sample from this child, and it was thus impracticable to further characterize these variants, particularly those predicted to disrupt the donor splice site. Nevertheless, these variants are very likely to lead to a disrupted CCDC40 protein, and consequently to dysfunction of the CCDC39-CCDC40 complex, which possibly justifies the observed axoneme patterns and the *situs inversus totalis*. Previous studies found that *CCDC40* pathologic variants are often of high impact (Tables S4 and S5) [34,35,36,37,38,39,40,41,42,43,44,45,46,47,48,49] and patients presented the worst clinical features [50]. Our data agree with these previous associations, as Patient-1 presented a heterogeneous pattern of cilia axoneme ultrastructural anomalies, higher variation in ciliary beat axis and ciliary deviation, high impact variants, and a more severe phenotype.

In Patient-2, the ultrastructural analysis of cilia axonemes revealed a homogeneous pattern of absence of both DAs and nexin bridges. This patient revealed two novel compound heterozygous variants in the *DNAH5* gene (frameshift and nonsense variants) and two rare compound heterozygous missense variants in the *DNAH7* gene.

*DNAH5* codes for a dynein heavy chain (DHC) that is localized on the ODA. It is a major disease-causing gene in PCD patients with ODA defects [43]. Both variants can potentially lead to a premature non-functional protein and both affect relevant domains of *DNAH5* (Figure S5). Particularly, the nonsense variant (inherited from the father) is in a hydrolytic ATP binding site of the dynein motor region D1 (or AA1), which is highly conserved [51]. It is the main ATPase site and its movement is critical for dynein function [22]. Likewise, the frameshift variant (inherited from the mother) is located at the DHC-N2 domain, which contains the neck region that is thought to mediate the power stroke [52]. Therefore, those variants were believed to justify the phenotype of Patient-2.

To gain more understanding of the molecular function of those novel *DNAH5* variants, we studied *DNAH5* mRNA expression and the location of the DNAH5 protein on nasal cells from the patient and his carrier parents. In both Patient-2 and his parents, a significant reduction of mRNA expression was observed. Furthermore, we noted a significant decrease in staining of the DNAH5 in cilia axonemes. In cells from the parents of the Patient-2, a cytoplasmic accumulation was observed, instead of distribution along the ciliary axoneme as observed in control individuals. Unfortunately, and due to ethical reasons, we did not have access to further samples from Patient-2 and his parents to perform western blot analyses. Those analyses would be important to infer the total protein amount, which would help to further comprehend the consequences of the presented variants. However, from IF intensity measurements, we can note that the decrease in staining of the DNAH5 in cilia axonemes from both parents was not fully balanced by the observed increase staining in the cytoplasm. This suggests that, besides the improper protein location, with an accumulation within the cytoplasm, the total amount of protein was also reduced. Previous reports also presented this same DNAH5 staining pattern in PCD patients with *DNAH5* mutations [27,43]. Moreover, these authors observed a different staining pattern of DNAH5 depending on the type of mutation, demonstrating that there are several, still largely unknown, mechanisms of the regulation of the protein trafficking. The correct subcellular localization of proteins is as important as the amount of functional protein produced for their function on cells and aberrant localization of proteins is implicated in the pathogenesis of several human diseases [53]. Further studies on protein location are vital in PCD patients, as it may help to bridge genotype and phenotype severity. Altogether, it gives an additional piece of evidence that both variants are causative and could justify the lack of ODA in our patient.

Previous works have described an association between *DNAH5* pathogenic variants and the absence of both DAs (Tables S4 and S5). However, null dnah5 mice showed normal IDA and absent ODA [54]. As Patient-2 showed the absence of both DAs, it is intriguing to speculate that alterations in another gene product could act together with *DNAH5*. Curiously, the *DNAH7* gene, in which we also find potentially disease-associated variants, is a member of the IDA family, which also belongs to the DHC. DNAH7 was already proved to be important for cilia motility [55,56]. It was found to be missing in a patient with PCD who had an absence of IDA and displayed abnormal cilia motility, but with normal ODA [56]. Both variants here found in the *DNAH7* gene affect highly conserved amino acids and are predicted to be disease-causing according to several bioinformatic tools (Table S3). Therefore, we suggested the possibility of the combination of *DNAH5* and *DNAH7* compound heterozygous variants may explain the Patient-2 phenotype, specifically the absence of ODA and IDA, respectively.

DHC belongs to the AAA+ superfamily of mechanochemical enzymes, with six AAA domains that assemble into a ring structure. One ring surface is covered by the N-linker (involved in dynein’s power stroke) and the opposite surface is covered by the C-terminal. DHC contains a stalk with an ATP-sensitive MTBS at its tip. MTBS is a small alpha-helical domain (H1-H6) at the end of the stalk, being responsible for binding the MT track. Alpha-helices H3 and H6 are major polar (H3 electrostatic surface shown in Figure 3b) and establish important contact points to the microtubule [57]. Alignment with cytoplasmic dynein proteins suggests that the *DNAH7* variant c.8209G < A (inherited from Patient-2 father), occurs at MTBS H3 (Figure 3e; Figure S5). The H3 sequence W2736XXXY2740 with Y highly polar (= K or R or Q), is conserved. W2736 is involved in an intermolecular interaction with the loop between helices H1 and H2 and Y2740 establish a salt bridge with the microtubule (Figure 3c) [57]. Nearly all of the MTBS residues involved in interactions with the microtubules (MT) are highly conserved, and mutating them results in defects in MT binding [58,59]. Further, glycine is a singular amino acid, because it contains hydrogen at its side chain and can play a distinct functional role, such as using its backbone to bind to phosphates and, consequently, changes in its conserved glycine are expected to be damaging [60]. It is; thus, plausible that the p.(Gly2737Ser) mutation, at MTBS alpha-helice H3, cause defects in protein function, likely by affecting the affinity towards MT. Regarding mutation, inherited by Patient-2 mother, p.(Arg3983Trp) is placed in a highly-conserved region at the C-terminal and, despite no direct structural or functional role being attributed to the residue, it is solvent exposed (Figure 3d, Figure S5). Arginine is a highly polar and positively-charged amino acid that is generally placed at the protein surface [60], whereas tryptophan is a nonpolar residue that is preferentially buried in protein hydrophobic cores. Therefore, this may affect protein stability. Further, according to the web server MUpro, both variants gave a negative ΔΔG, which corroborates the prediction of decreasing the protein stability [61].

Additionally, to get further knowledge about the functional role of *DNAH7* variants, we studied *DNAH7* mRNA expression and protein location. It was observed a reduced mRNA expression in Patient-2 nasal cells, but no significant change was observed in his parents.

Interestingly, it was observed a reduction in the staining of DNAH7 protein in cilia axonemes and an accumulation of the DNAH7 staining within the cytoplasm in Patient-2 and his father, but not in his mother, which presented a typical location along the ciliary axoneme. The increase in cytoplasmic staining was more evident in the father. In contrast to what we observed from IF analyses in DNAH5, decreased staining in DNAH7 in cilia axonemes from the father appeared to be balanced by the increased staining in the cytoplasm. This suggests that, in this case, there is not a protein reduction in the father of Patient-2, but an improper localization, with protein accumulation within the cytoplasm. Moreover, this may suggest that the alpha-helice H3 (where the variant presented by the father is placed) may be important for proper protein addressing/function and suggests the variant is pathogenic. This agrees with our bioinformatic predictions (Table S3), which predicted that this very rare *DNAH7* variant (p.(Gly2737Ser)) potentially will disrupt the protein stability and, given its important localization on the microtubule binding site region, could reduce the affinity of dynein to microtubule and, consequently, affect the function. In contrast, in regards to the *DNAH7* variant inherited from the Patient-2 mother, although bioinformatic tools predicted the variant as potentially damaging (as above explained), this variant has a slight high population frequency and the domain where it is localized has no structural or functional role attributed. In agreement, the variant alone may not be harmful enough as the results observed in the mother of Patient-2 confirmed, which showed a staining pattern identical to control individuals. Further, it may suggest that the C-terminal may not be critical for protein targeting. However, in Patient-2 the combination of both *DNAH7* variants are expected to contribute to potentiate the effect leading to the absence of patient IDA.

In the parents of Patient-2, particularly in his father, the wild-type DNAH5 and DNAH7 proteins would be in too low amounts to be detected in the IF analyses. However, the remaining, correctly-localized, wild-type proteins present in the cells may be sufficient to ensure cilia beating and a normal phenotype. Furthermore, the differences observed among our studied patient and his parents may be likely the consequence of the combination of an individual’s coding gene variants (i.e., the combination of *DNAH5* and *DNAH7* variants) that influences the phenotype in an individual-specific way [62]. This agrees with the recessive pattern of the disease and demonstrates that the parents are asymptomatic carriers of single-mutant alleles, which only cause disease when in homozygosity or compound heterozygosity. Further, it can demonstrate the existence of highly regulated modes of translation and/or protein-degradation regulation that differ in a single heterozygous and in compound heterozygous. Further, we highlight the need for further studies that analyze the correlation between mRNA expression levels, protein function, and the severity of the phenotype. A study from Ruzycki et al. (2015), using mice models of retinopathies (another ciliopathy as is PCD), demonstrated that the correlation of gene expression changes with phenotype severity is not linear nor straightforward [63]. The authors showed the existence of thresholds in gene expression that determined the phenotypic presentation. For instance, they reported the case of two mice that have drastically different phenotypes but have only marginal differences in gene expression. As far as we know, no similar study was performed in PCD. A similar study with PCD animal models would be of upmost importance. It would help to understand the high panoply of phenotypes displayed by PCD patients and likely could explain the results here presented.

To the best of our knowledge, we were the first report to show immunofluorescence localization of DNAH7 protein in ciliary cells and to report a potentially pathogenic variant in the *DNAH7* gene with an association to the PCD/KS phenotype. Further, no study has shown the interaction of two genes in the PCD phenotype. Considering the information given by previous knockout models and PCD complexity and heterogeneity, we hypothesize that an effect of *DNAH7* variants over the phenotype may exist. Either genes’ products when defective may have; thus, caused the absence of both DAs in Patient-2. The variants in gene *DNAH5* are potentially more damaging and are, thus, expected to be the major factor responsible for Patient-2 phenotype. Nevertheless, *DNAH7* may have an accumulative effect, leading to the complete loss of both DAs. Our functional data corroborates these assumptions, as we observed a reduction of gene expression and protein mislocalization in both genes. Even though, as it occurs for all potential pathogenic variants, more accurate evidence of the genotype–phenotype correlation will only be possible using gene knock-in animal models. In 2013, the type II bacterial CRISPR/Cas9 system brought the promise to revolutionize genome editing [64,65]. Nonetheless, knock-in animal models are still difficult to obtain and protocols attempting to improve the efficiency rate are still being published. The major limitations are related to the need for homologous recombination to mediated gene knock-in. Although homologous recombination is well-established, it is not applied in every cell type in the same way due to the variable homologous recombination frequencies, which, together with the larger size of our genes, makes studies with gene knock-in difficult. Further, it is important to highlight that, even though animal models are critical to unveil genotype–phenotype correlations, caution must be used as those models do not always represent the full range of human phenotypes. A specific example was given by cystic fibrosis mice models attempting to explain the ΔF508 mutation, the more frequent mutation found in cystic fibrosis. In these animal models, the pulmonary pathophysiological changes observed in humans were not observed in mutated mice, which did not show significant lung pathology [66]. Consequently, works showing particular pieces of evidence must be taken into account to increase the genotype–phenotype knowledge of these complex diseases. If the hypotheses here presented were proven to be realistic, it would certainly increase the accuracy of the genotype–phenotype correlations in PCD.

WES is undoubtedly powerful and a determinant for the discovery of new genetic causes of diseases. The possibility of multiple variants in different genes contributing to the same phenotype opens a new door in genomic research and brings an additional important advantage of applying WES or multi-gene NGS panels over the (single) gene-by-gene approach of Sanger sequencing. Additional studies are pivotal to understand the effects of variants in multiple genes on the PCD phenotype. Further, it is also critical to increase our knowledge regarding the biological mechanisms that control the regulation of mRNA levels and protein abundances/degradation, as well as the co-translational and post-translational mechanisms that regulate protein location. Nowadays, the main challenge placed in complex genetic diseases, such as PCD, is not only to know which genes and variants are associated with the phenotype, but also to obtain refined genotype–phenotype correlations, for each variant and the different genetic factors, explaining why two individuals sharing the same genotype or similar variants may develop dissimilar phenotypes. Generating that knowledge would be critical to unveil the complexity of PCD, as well as their implications in reproductive function and fertility.

## 5. Conclusions

In conclusion, in Patient-1 we identified two compound heterozygous variants in the *CCDC40* gene, one affecting the splicing region, not indicated in ClinVar, and one frameshift, previously indicated in ClinVar, both here firstly reported. This indicates that, although already described as a common PCD gene, new variants are still to be reported, which creates difficulty in standard genetic diagnosis. In relation to Patient-2, we identified four compound heterozygous variants; two novel in the *DNAH5* gene, one frameshift, and another nonsense; and two in the *DNAH7* gene, both missense and not indicated in ClinVar, all here firstly reported. Functional studies revealed that in Patient-2 both genes presented reduced mRNA expression and that the protein is significantly reduced from cilia. These observations draw attention to the possibility of the interaction of these two genes in the patient phenotype. This manuscript highlights the need for further studies that validate the hypothesis presented, in which gene interactions and/or modifier genes are possibly a major cause of the high phenotypic variability observed in these patients.

## Figures and Tables

**Table 1:** List of pathogenic variants found in Patient-1 and Patient-2.

Patient	Gene	Variant Description	Freq.*	Variant Origin
Patient-1	*CCDC40*	NG_029761.1(NM_017950.3): c.1989 + 1G > A; p.(=)	0.0008%	P
		NM_017950.3: c.2824_2825insCTGT; p.(Arg942Thrfs*57)	0.0042%	M
Patient-2	*DNAH5*	NM_001369.2: c.4530del; p.(Asn1511Metfs*6)	New	M
		NM_001369.2: c.6000C > A; p.(Tyr2000*)	New	P
	*DNAH7*	NM_018897.3: c.8209G > A; p.(Gly2737Ser)	0.0008%	P
		NM_018897.3: c.11947C > T; p.(Arg3983Trp)	0.8666%	M

M = maternal origin; P = paternal origin; * = from Exome Aggregation Consortium (ExAC) and dbSNP databases.

## Supplementary Materials

The following are available online at https://www.mdpi.com/2073-4409/8/8/900/s1. Figure S1: Ultrastructure of cilia axoneme, Figure S2: Genetic analysis of Patient-1, Figure S3: Representation of multiple protein sequence alignment of the variants p.Gly2737Ser (c.8209G > A) and p.Arg3983Trp (c.11947C > T) found in the *DNAH7* gene, Figure S4: Genome-wide homozygosity mapping using WES data and the HomozygosityMapper software. Figure S5. The architecture of the dynein motor. Table S1: Exome-sequencing quality metrics; Table S2: List of primers used in both conventional polymerase chain reaction (PCR) and real-time quantitative PCR (qPCR) of this study. Table S3: Bioinformatic analysis of DNAH7 variants found in Patient-2. Table S4: Compilation of published variants in *CCDC40* gene. Table S5: Compilation of published variants in *DNAH5* gene.
