# Supplementary material for: Clinical and Genetic Analysis of Children with Kartagener Syndrome
Source: Cells. 2019 Aug 15;8(8):900. doi: 10.3390/cells8080900 (PMC6721662; doi:10.3390/cells8080900)
Supplement: Supplementary file 1 [file cells-08-00900-s001.zip › cells-546194-supplementary/Supplementary Figure S4.docx]

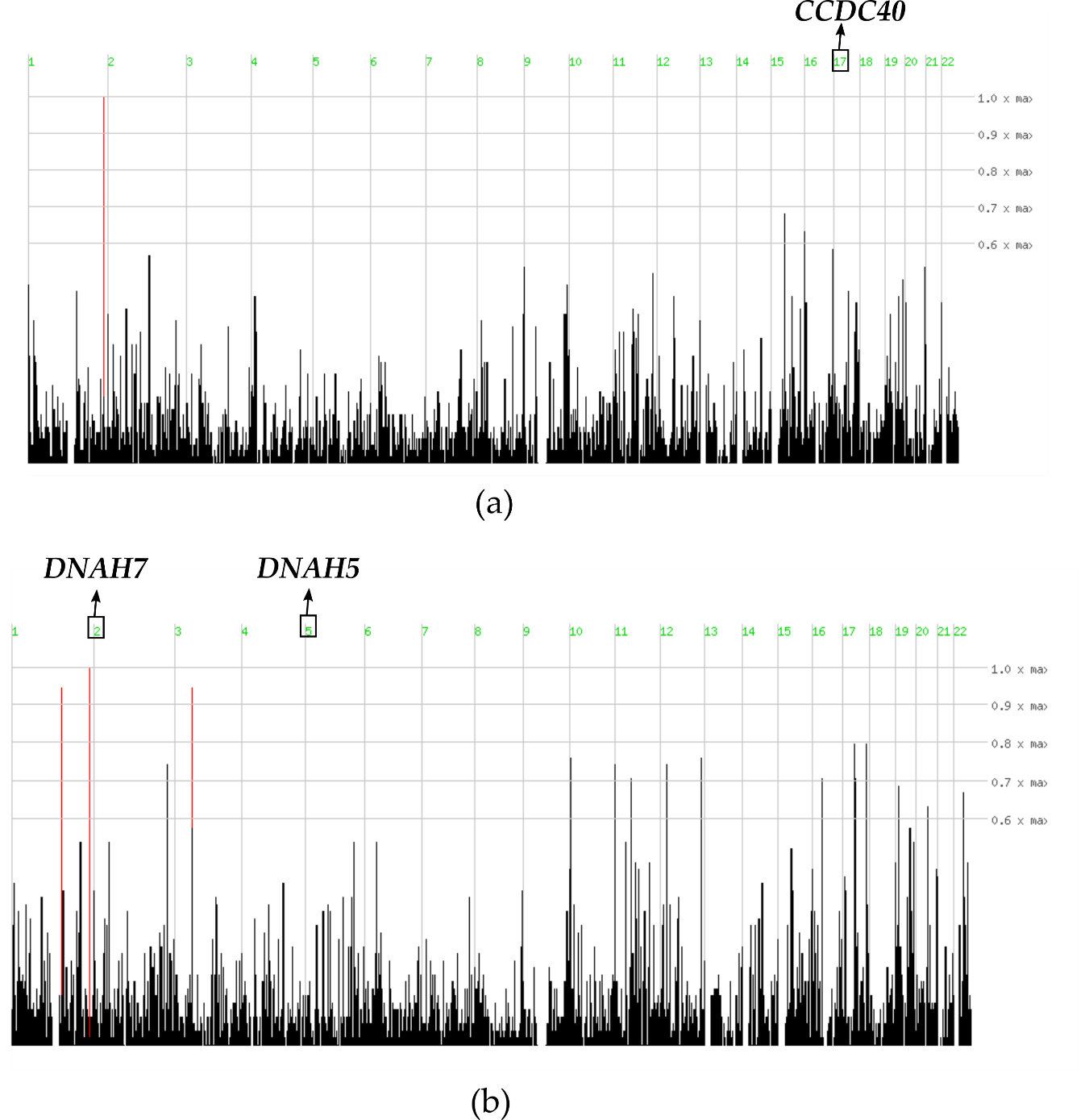


**Supplementary Figure S4.** Genome-wide homozygosity mapping using WES data and the HomozygosityMapper software (<http://www.homozygositymapper.org/>). Homozygosity mapping from (**A**) Patient 1 and (**B**) Patient 2. In both cases, just a small homozygous region in chromosome 1 (A) and 1 and 3 (B) were identified (highlighted in red). Longer stretches of homozygous segments were not identified. Only a small homozygous region in chromosome 1 (Patient 1) and 1 and 3 (Patient 2). None of these stretches were in the chromosome where the causative genes were identified. Homozygosity mapping relies on the assumption that an affected individual inherits two identical alleles of a disease gene from a common ancestor. It is typically applied in small, closed/consanguineous population [30]. Notwithstanding, we previously showed statistically significant differences after the correlation between two parameters obtained with the HomozygosityMapper software between individuals whose disease-causing variants were homozygous and individuals presented compound heterozygous variants [31]. This corroborates the presence of compound heterozygous autosomal recessive variants in our patients.

**Supplementary references***

30. Oliveira, J.; Pereira, R.; Santos, R.; Sousa, M. Homozygosity Mapping using Whole-Exome Sequencing: A Valuable Approach for Pathogenic Variant Identification in Genetic Diseases. In Proceedings of 10th International Joint Conference on Biomedical Engineering Systems and Technologies. BIOINFORMATICS. ; pp. 2010-2016.<https://doi.org/10.5220/0006248502100216>.

31. Oliveira, J.; Pereira, R.; Santos, R.; Sousa, M. Evaluating Runs of Homozygosity in Exome Sequencing Data - Utility in Disease Inheritance Model Selection and Variant Filtering. In *Communications in Computer and Information Science*, Barbosa, S.D.J., Chen, P., Filipe, J., Kotenko, I., Sivalingam, K.M., Washio, T., Yuan, J., Zhou, L., Eds. Springer, Cham: Berlin, Germany, 2018; Vol. 881, pp. 268-288.<https://doi.org/10.1007/978-3-319-94806-5_15>

*reference numbers as appear in the main text
