# Supplementary material for: Clinical and Genetic Analysis of Children with Kartagener Syndrome
Source: Cells. 2019 Aug 15;8(8):900. doi: 10.3390/cells8080900 (PMC6721662; doi:10.3390/cells8080900)
Supplement: Supplementary file 1 [file cells-08-00900-s001.zip › cells-546194-supplementary/Supplementary Figure S5-revised.docx]

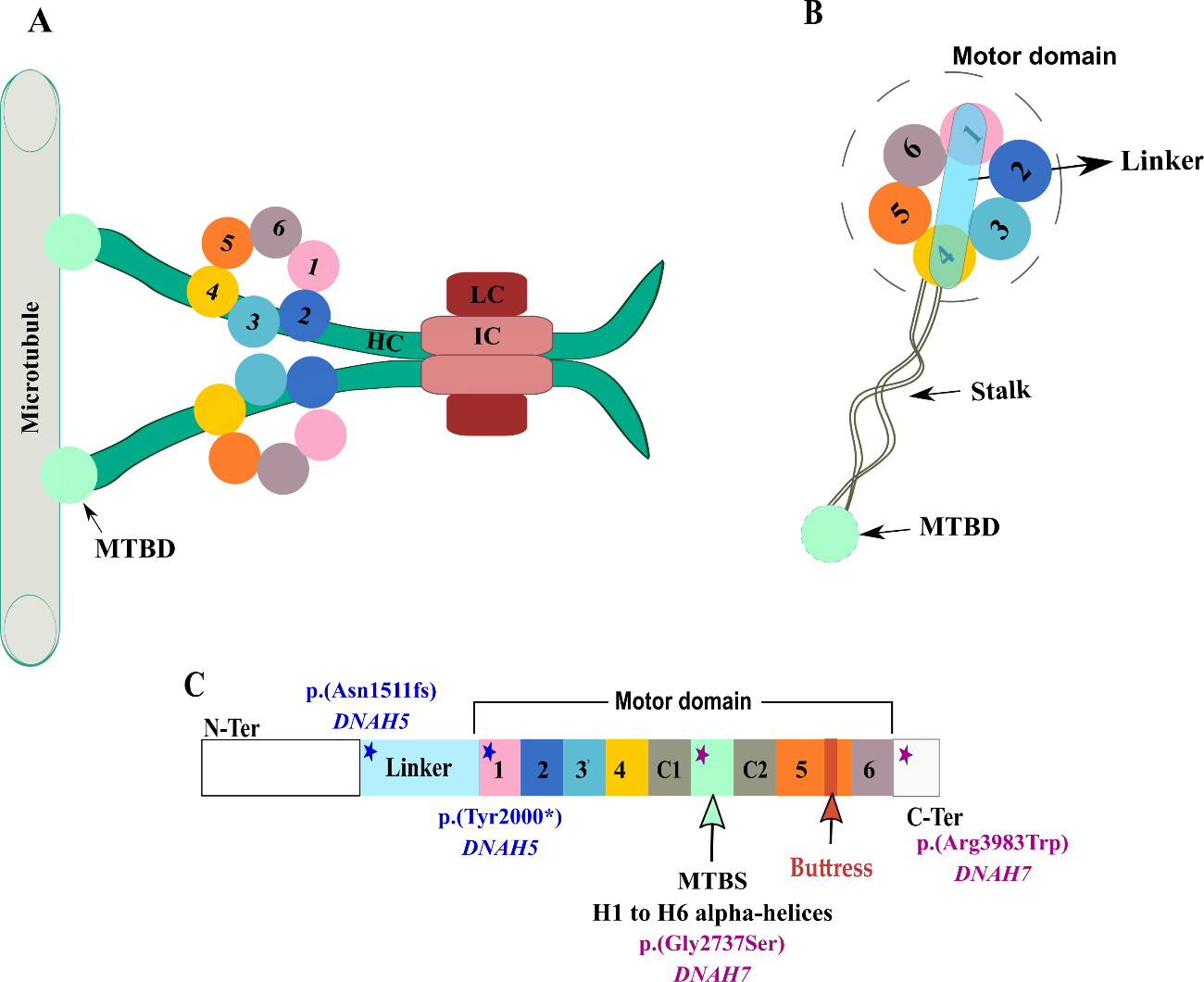


**Figure S5. The architecture of the dynein motor. (A)** Schematic representation of a dynein motor complex. Dynein motor is a dimer containing two identical heavy chains (HC) subunits, with its motor domain and microtubule binding site (MTBS), and intermediate and light chains, respectively IC and LC. **(B)** Diagram representing dynein heavy chain domain structure: the dynein motor domain with its ring of six AAA domains (numbers 1 to 6 represent, correspondingly the AAA1 to AAA6); the linker (or neck ) domain, that stretches across a ring of six AAA domains; the stalk, which emerges from the small domain of AAA4 and ends with the MTBS. **(C)** The illustration of the linear map of a general dynein HC composition, including the N-terminal tail, the linker and the six AAA domains (labelled 1–6). The position of the stalk is represented in dark green, by the two predicted coiled coil structures CC1 (C1) and CC2 (C2) that are located between AAA4 and AAA5. MTBS (that contains alpha-helices H1 to H6) and buttress are also indicated, as well as the C-terminal domain. Here are also represented the place where the variants from Patient 2 in both *DNAH5* (blue) and *DNAH7* (purple) gene are located. Adapted from [23, 52].
