# Supplementary material for: Clinical and Genetic Analysis of Children with Kartagener Syndrome
Source: Cells. 2019 Aug 15;8(8):900. doi: 10.3390/cells8080900 (PMC6721662; doi:10.3390/cells8080900)
Supplement: Supplementary file 1 [file cells-08-00900-s001.zip › cells-546194-supplementary/Supplementary Figures S1-revised.docx]

**Supplementary Figures S1A**

**Controls**


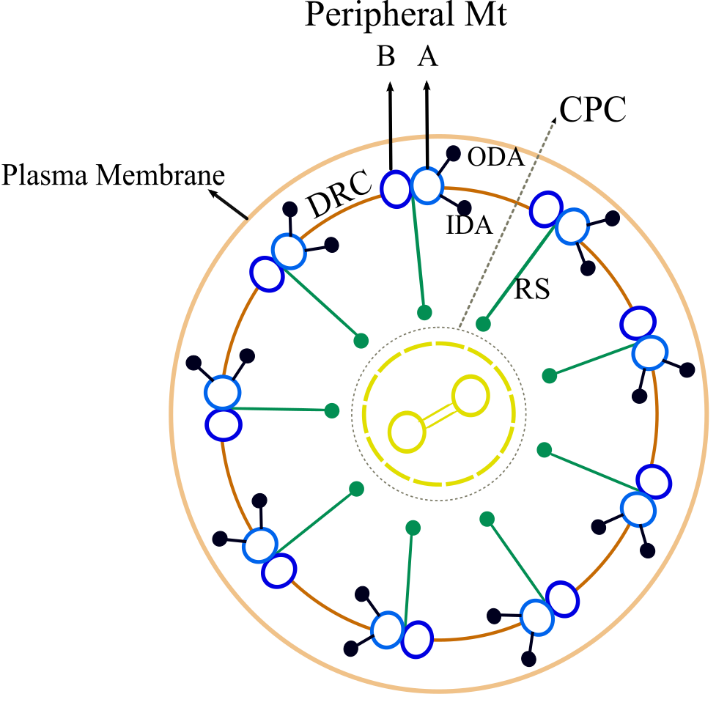


**Schematic representation of normal cilia axoneme**. There are nine peripheral doublets made of joined complete microtubule A and incomplete microtubule B. Dynein arms, inner (IDA) and outer (ODA), are expansions of the microtubule A of the nine peripheral doublets. The central pair (CP) is linked to the peripheral doublets by the radial spokes (RS). Peripheral doublets are interlinked by nexin bridges (nx).


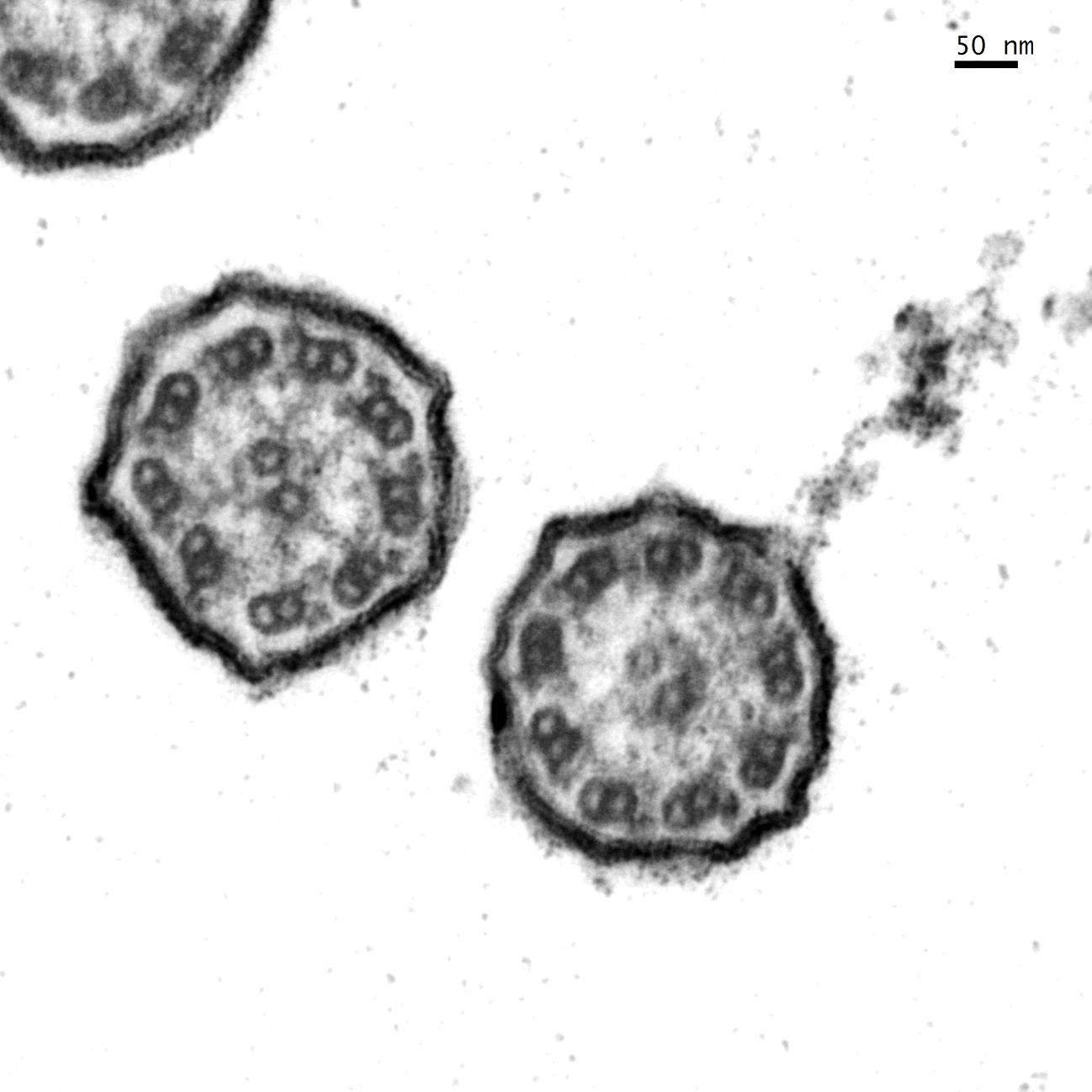


**A**

**B**

**RS**

**ODA**

**IDA**

**CP**

**RS**

**i**

**i**

**i**

**i**

**i**

**i**

**i**

**i**

**o**

**o**

**o**

**o**

**o**

**o**

**o**

**o**

**nx**

**nx**

**nx**

**nx**

**nx**

**nx**

**nx**

**nx**

**nx**

**Cilia axoneme from control individuals**. There are nine peripheral doublets made of joined complete microtubule A and incomplete microtubule B. **Dynein arms**, inner (IDA; i) and outer (ODA; o), **are** **short and thick** expansions of the microtubule A of the nine peripheral doublets. **Note that, in the same plan, dynein arms with classical aspect are not observed in all doublets, especially the IDA**. The central pair (CP; circle) is linked to the peripheral doublets by the radial spokes (RS). Peripheral doublets are interlinked by nexin bridges (nx). **Nexin bridges frequently do not appear well defined**.


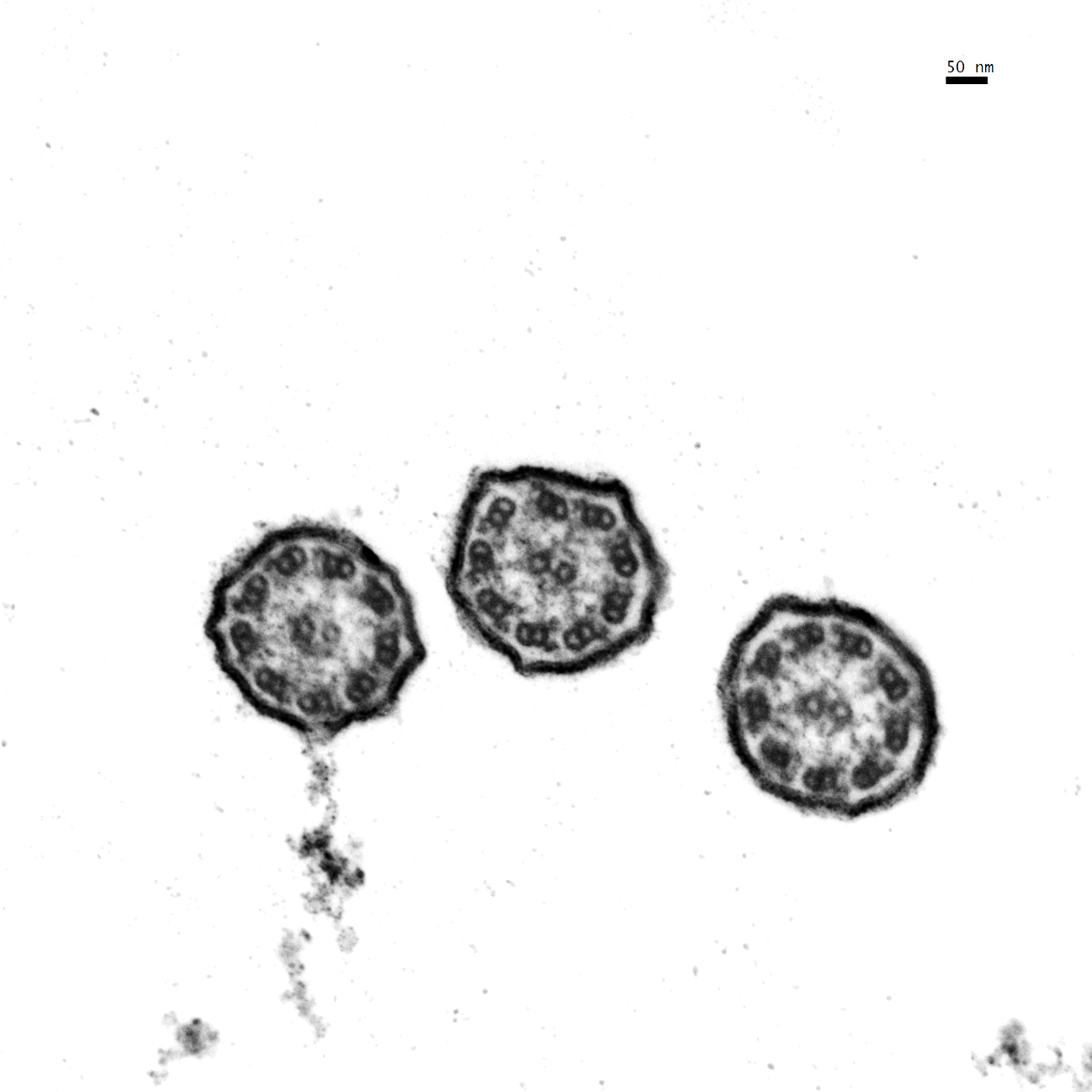


**i**

**i**

**i**

**i**

**i**

**i**

**i**

**i**

**i**

**o**

**o**

**o**

**o**

**o**

**o**

**o**

**o**

**o**

**RS**

**RS**

**nx**

**nx**

**nx**

**nx**

**nx**

**nx**

**nx**

**nx**

**nx**

**A**

**B**


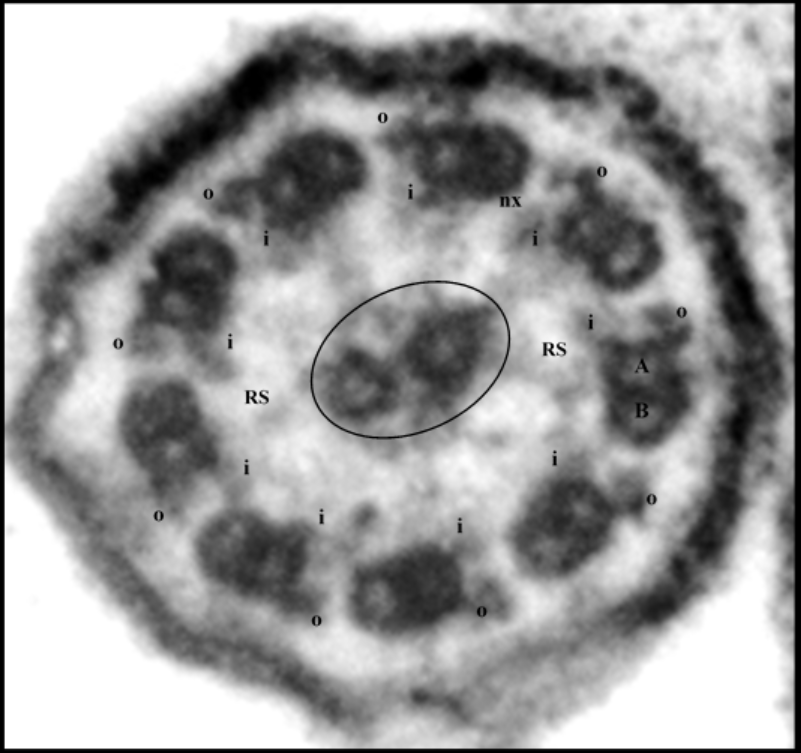


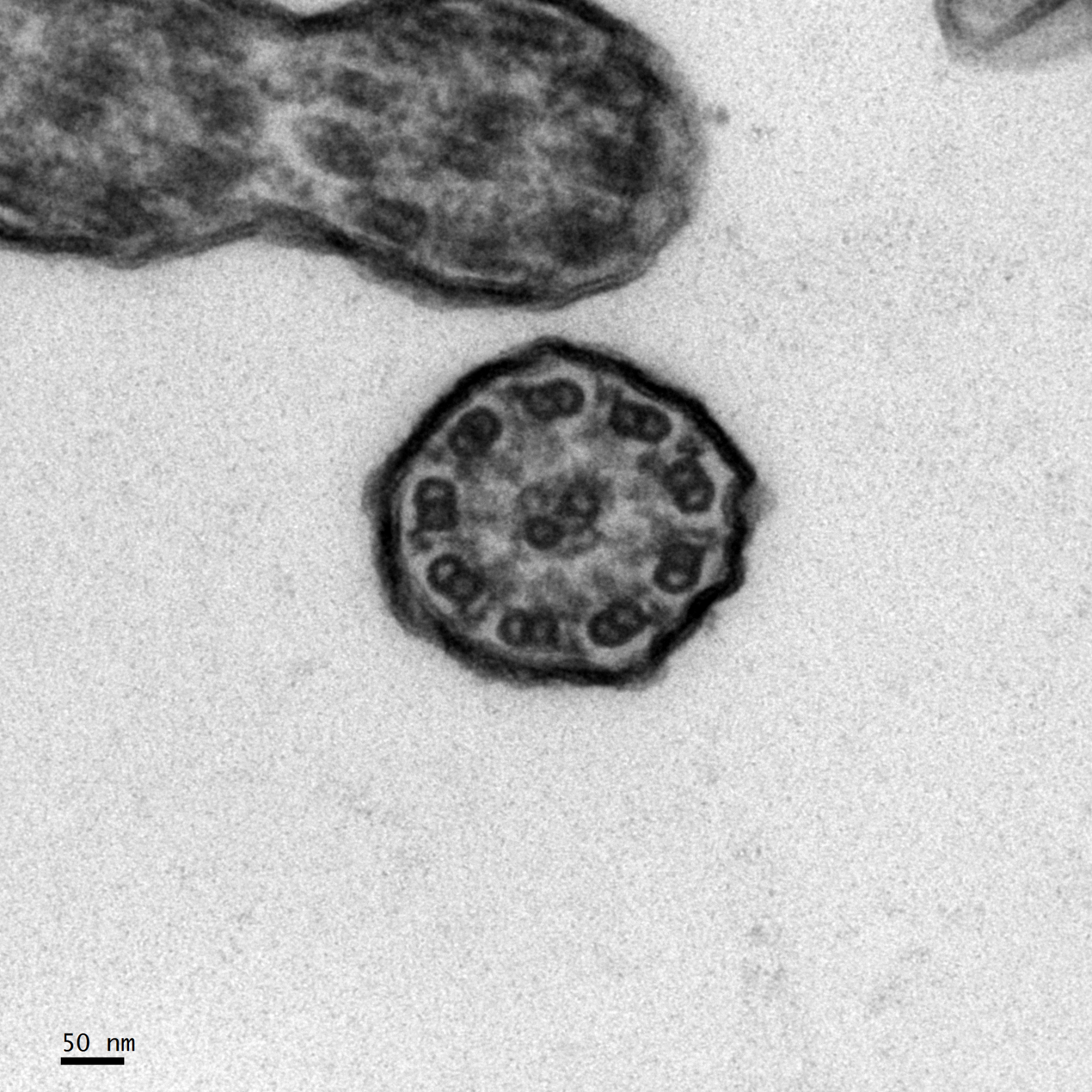


**nx**

**i**

**i**

**i**

**i**

**i**

**i**

**i**

**i**

**i**

**o**

**o**

**o**

**o**

**o**

**o**

**o**

**o**

**o**

**RS**

**RS**

**A**

**B**


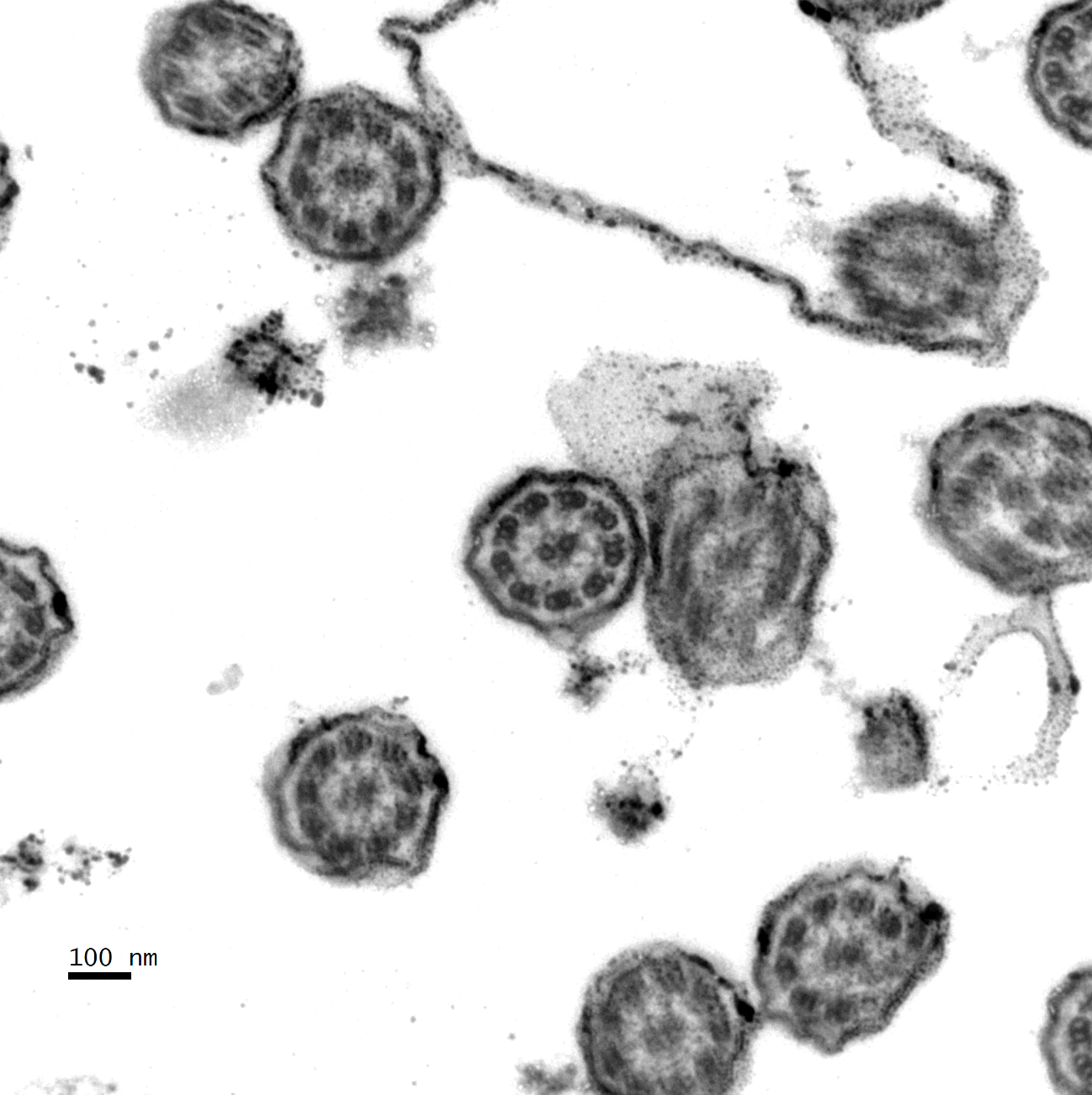


**RS**

**RS**

**i**

**i**

**i**

**i**

**i**

**i**

**i**

**i**

**o**

**o**

**o**

**o**

**o**

**o**

**o**

**o**

**o**

**i**

**A**

**B**

Slightly tilted (out of focus) image to better define dynein arms.

**Supplementary Figure S1B**

**Images from Patient 1 axoneme**

**B**

**o**

**o**

**o**

**o**

**o**

**o**

**o**

**RS**

**nx**

**nx**

**nx**

**nx**

**A**

**Patient-1**: absence of the inner dynein arms (dotted lines in doublets) with presence of the outer dynein arms (o) in seven doublets; absence of both dynein arms (dotted lines in doublets) in two doublets; absence of nexin bridges (nx: note that spaces between doublets are blank); displacement of the central pair (circle); several radial spokes (RS) are missing (dotted line between the central pair and doublet). Doublets have a microtubule A with dynein arms and a microtubule B.


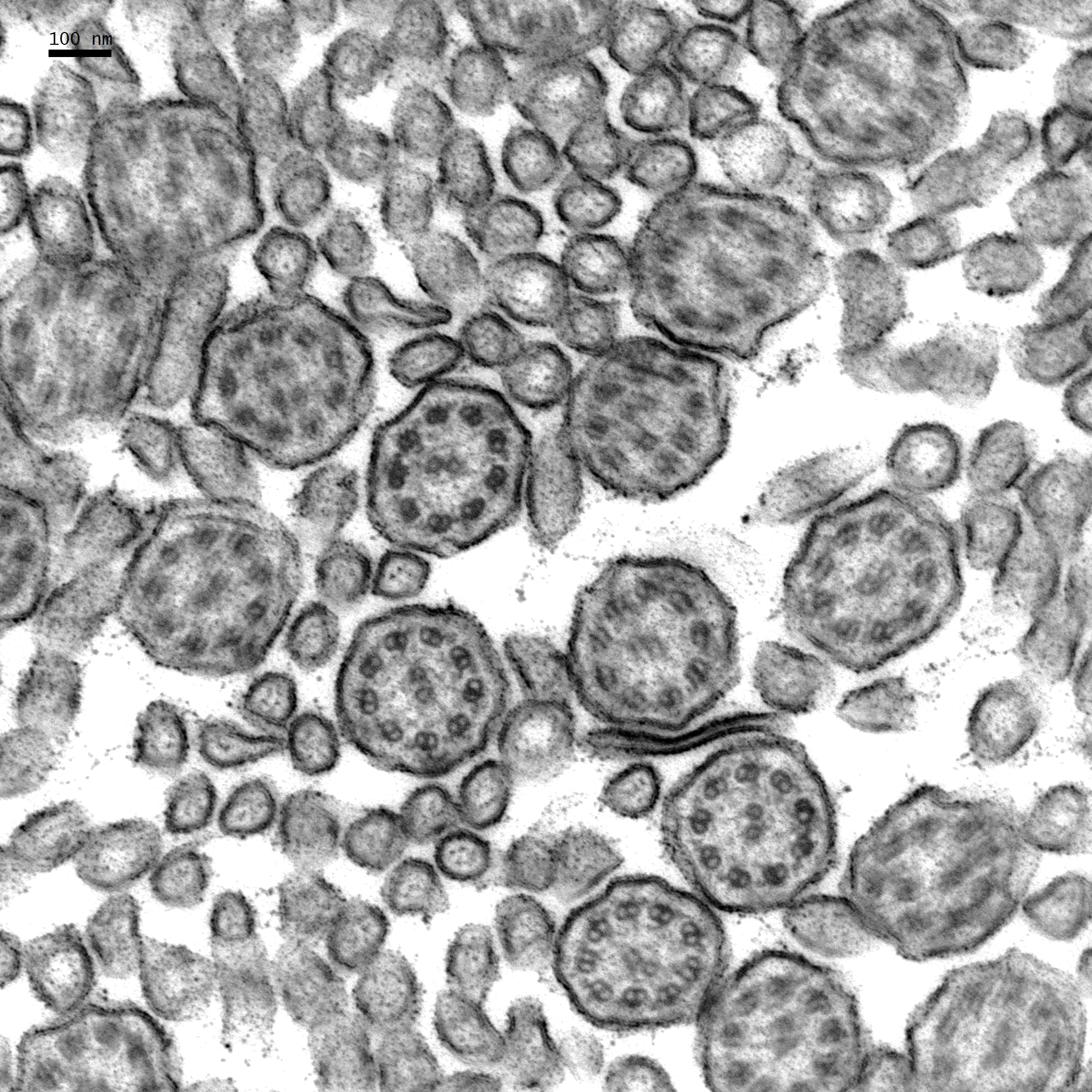


**o**

**o**

**o**

**o**

**o**

**o**

**o**

**i**

**nx**

**nx**

**nx**

**RS**

**A**

**B**

**Patient-1**: absence of both dynein arms (dotted lines in doublets) in two doublets; absence of the inner dynein arms with presence of underdeveloped outer dynein arms (o) in six doublets; presence of both dynein arms (i, o) in one doublet; absence of nexin bridges (nx). The central pair (circle) is slightly displaced. The radial spokes (RS) are mostly undefined (dotted line between the central pair and doublet). Doublets have a microtubule A with dynein arms and a microtubule B.


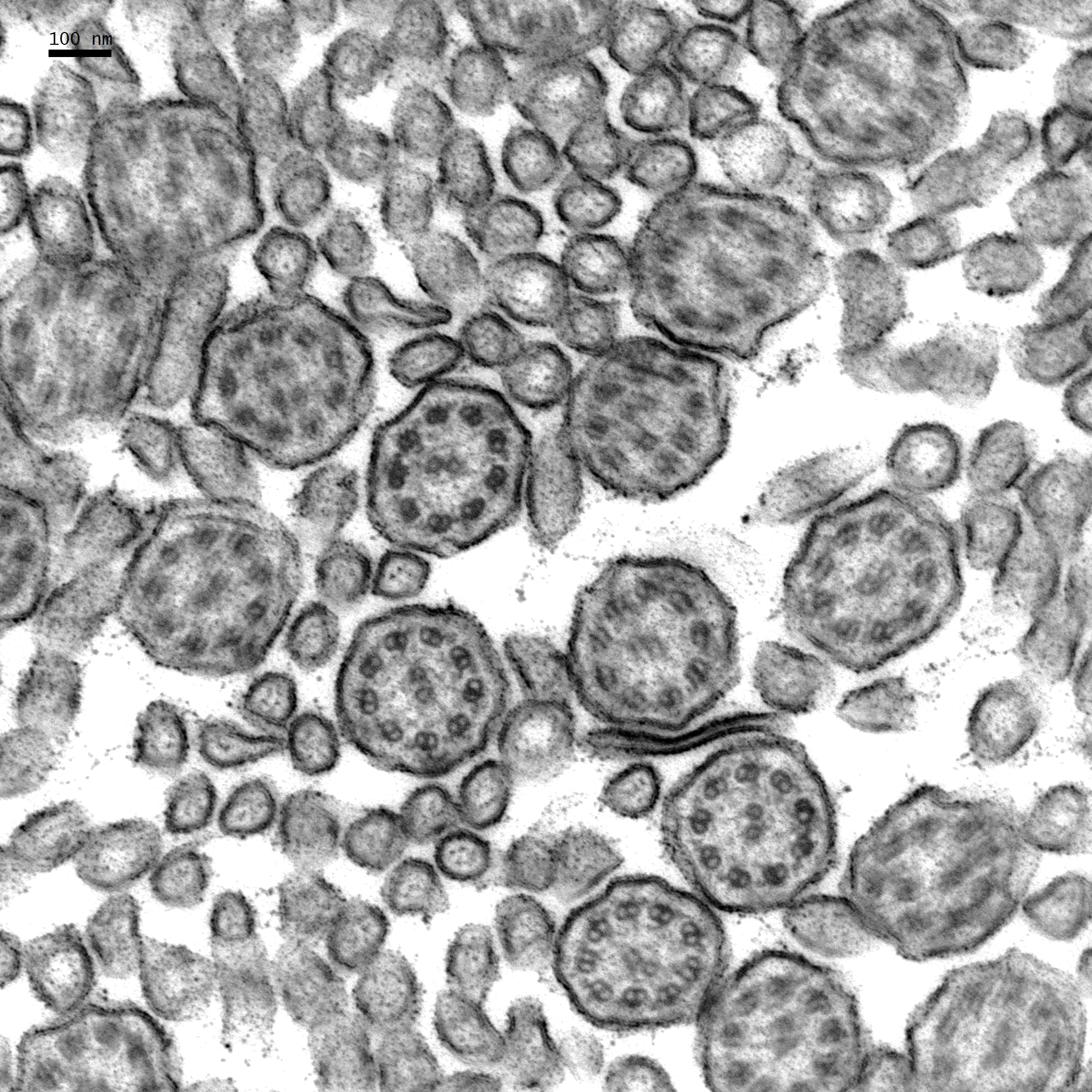


**nx**

**nx**

**o**

**o**

**o**

**o**

**o**

**o**

**o**

**o**

**i**

**RS**

**nx**

**A**

**B**

**Patient-1**: absence of both dynein arms (dotted lines in doublets) in one doublet; absence of the inner dynein arms with presence of underdeveloped outer dynein arm (o) in seven doublets; presence of underdeveloped both dynein arms (i, o) in one doublet; absence of nexin bridges (nx). There are doublets with nexin links (lines between doublets). The central pair (circle) is slightly displaced. The radial spokes (RS) are mostly undefined (dotted line between the central pair and doublet). Doublets have a microtubule A with dynein arms and a microtubule B.


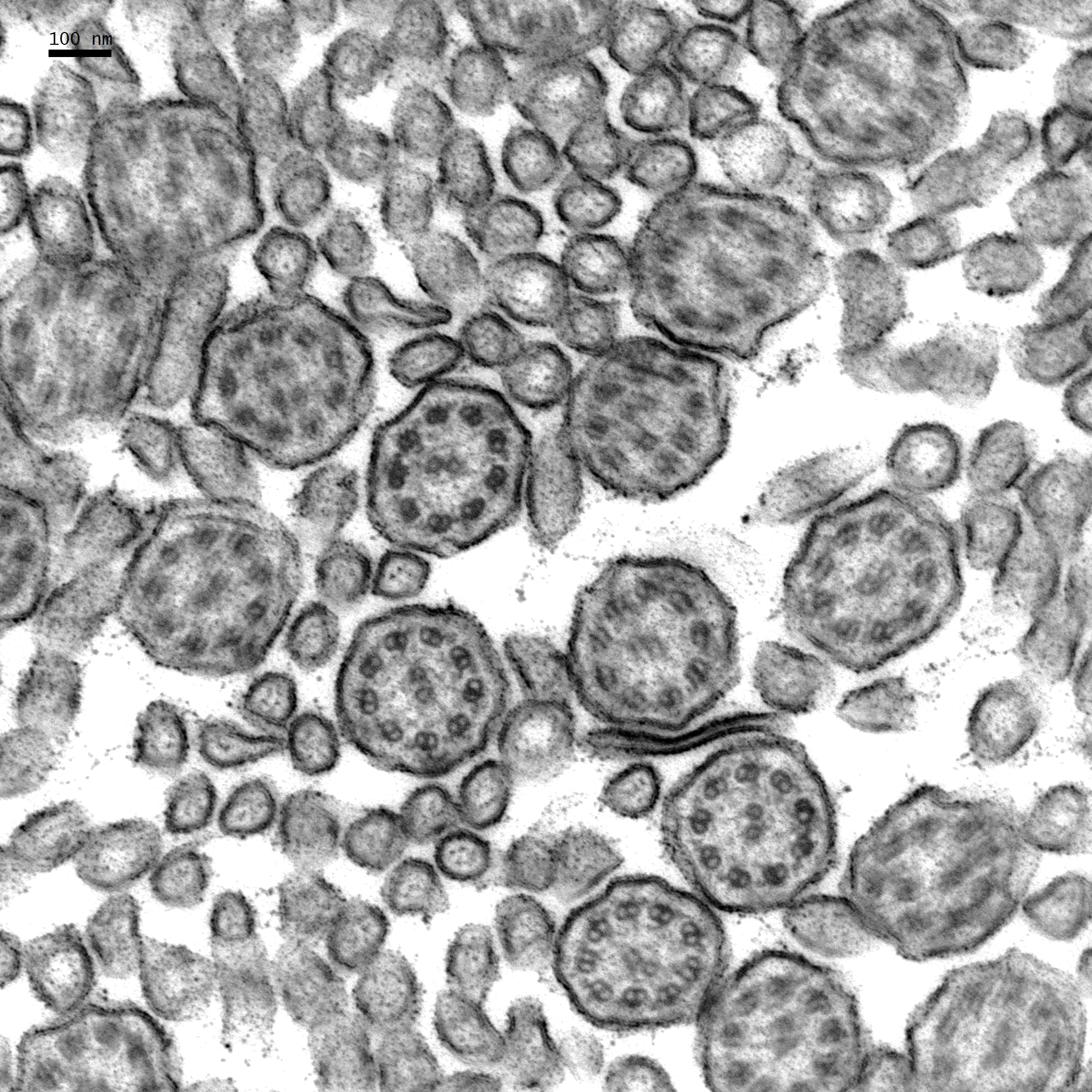


**nx**

**nx**

**o**

**o**

**o**

**o**

**o**

**o**

**o**

**A**

**B**

**Patient-1**: absence of the inner dynein arms (dotted line in doublets) with presence of underdeveloped outer dynein arm (o) in seven doublets; absence of both dynein arms (dotted lines in doublets) in two doublets; absence of nexin bridges (nx). The central pair (circle) is slightly displaced. The radial spokes are mostly undefined (dotted line between the central pair and doublet). Doublets have a microtubule A with dynein arms and a microtubule B.

**nx**

**nx**

**CP**

**D**

**o**

*****

**CP**

**nx**

**o**

**o**

**o**

**o**

**o**

*****

*****

*****

*****

*****

**nx**

**C**

**nx**

**nx**

**o**

**o**

**o**

*****

*****

*****

**CP**

**C**

**B**

**o**

**o**

**o**

**o**

*****

*****

*****

*****

**nx**

**nx**

**CP**

**CP**

**A**

**B**

**A**

**B**

**A**

**B**

**A**

**Patient-1**: **A**. absence of the inner dynein arms (*) with presence of the outer dynein arm (o) in four doublets; absence of both dynein arms in the other five doublets; absence of nexin bridges (nx). Presence of two central pairs (CP). Radial spokes are undefined. Doublets have a microtubule A with dynein arms and a microtubule B.

**B**. absence of the inner dynein arms (*) and presence of the outer dynein arms (o) in three doublets, with absence of both dynein arms in the other six doublets; absence of nexin bridges (nx). Displaced central pair (CP) with presence of a single microtubule of a second central pair (C). Radial spokes are undefined. Doublets have a microtubule A with dynein arms and a microtubule B.

**C**. absence of the inner dynein arms (*) and presence of the outer dynein arms (o) in six doublets, with absence of both dynein arms in the remaining doublets; absence of nexin bridges (nx). Displaced central pair (CP). Radial spokes are undefined. Doublets have a microtubule A with dynein arms and a microtubule B.

**D**. Dynein arms and radial spokes are undefined; absence of nexin bridges (nx). The central pair is displaced. One doublet is centrally displaced (arrow). Radial spokes are undefined.


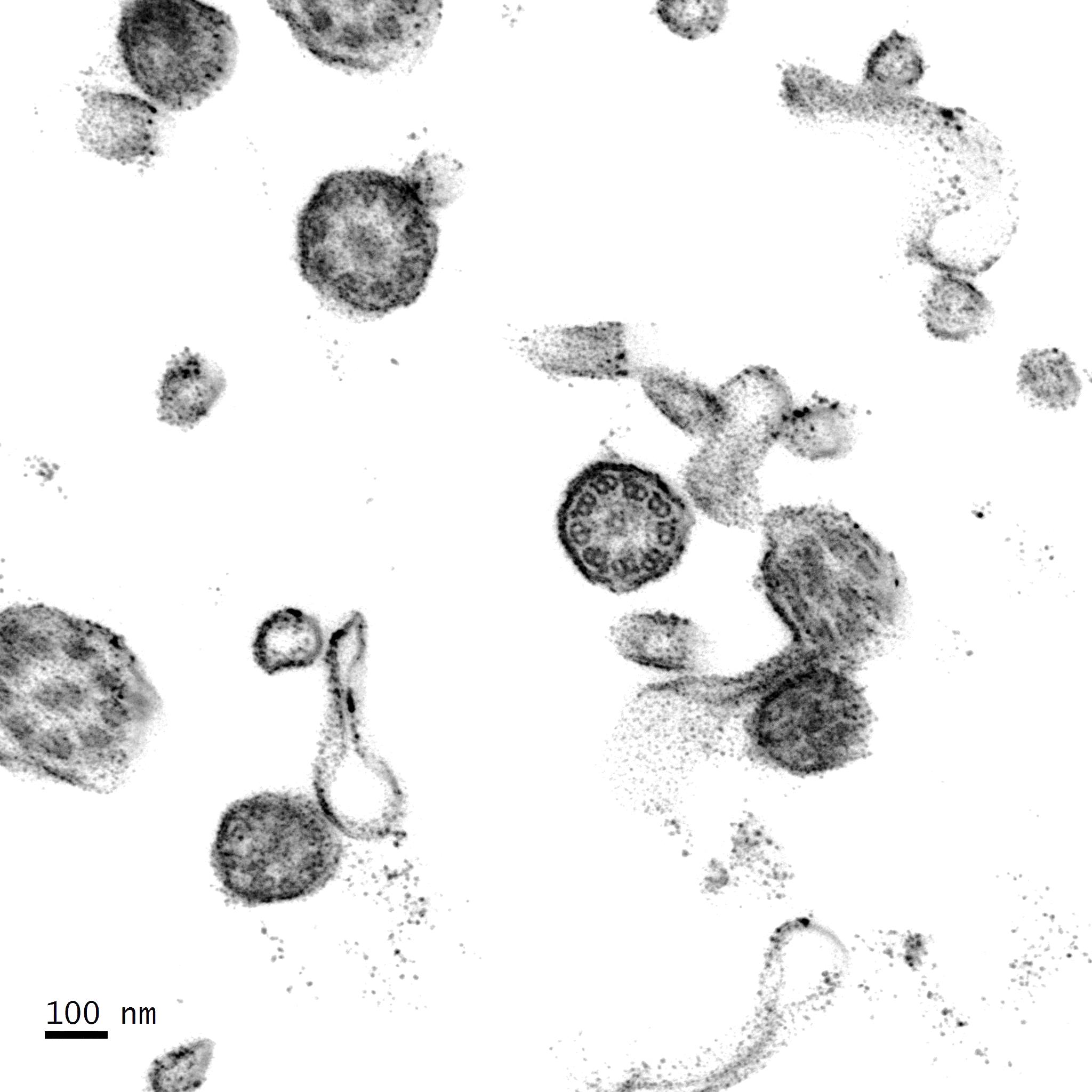


**C**

**nx**

**A**

**nx**


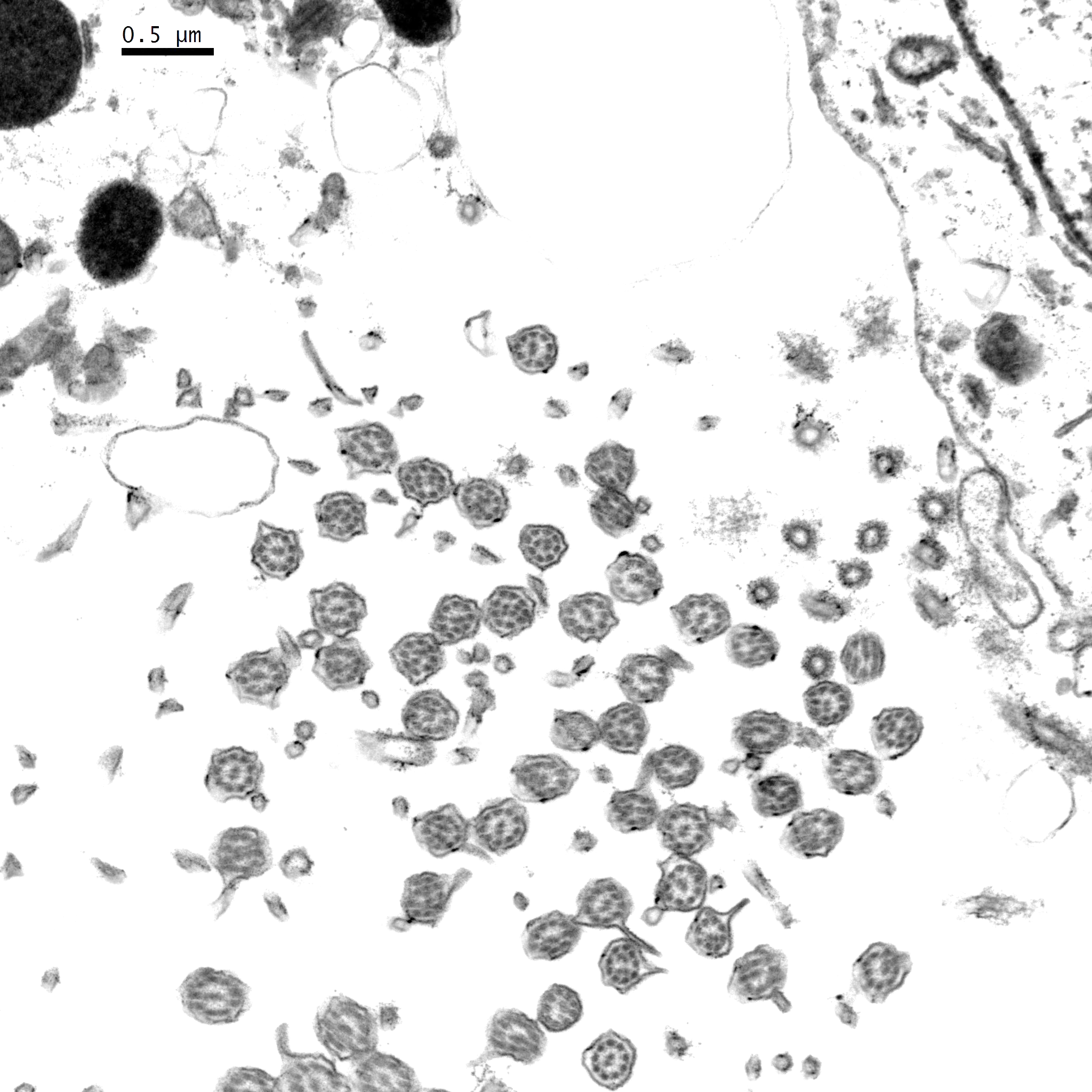


*****

*****

*****

**B**

**Patient-1**: **A**. presence of a single undefined central pair (C). Dynein arms and radial spokes are undefined. Absence of nexin bridges (nx). **B**. disruption (*) of the axoneme architecture.

**Supplementary Figure S1C**

**Images from Patient 2 axoneme**


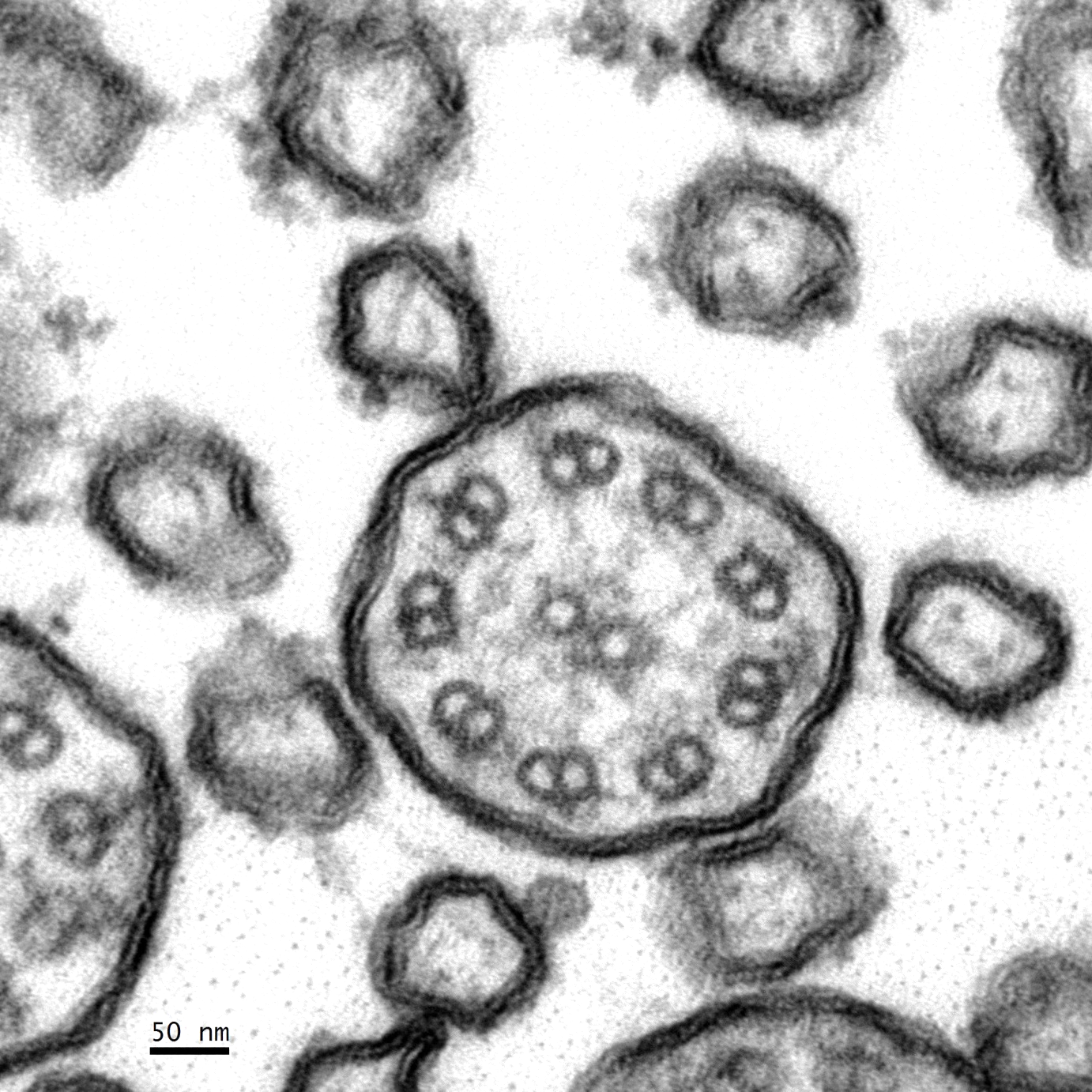


**A**

**B**

**Patient-2**: Absence of both dynein arms (dotted lines in doublets) and nexin links (dotted lines exemplified between some doublets). Normal presence of the central pair (circle) and radial spokes (black line exemplified between the central pair and doublet). Doublets have a microtubule A with dynein arms and a microtubule B.

**A**

**B**

**Patient-2**: Absence of both dynein arms (dotted lines exemplified in some doublets) and nexin links (dotted lines exemplified between some doublets). Normal presence of the central pair (circle) and radial spokes (black line exemplified between the central pair and doublet). Doublets have a microtubule A with dynein arms and a microtubule B.


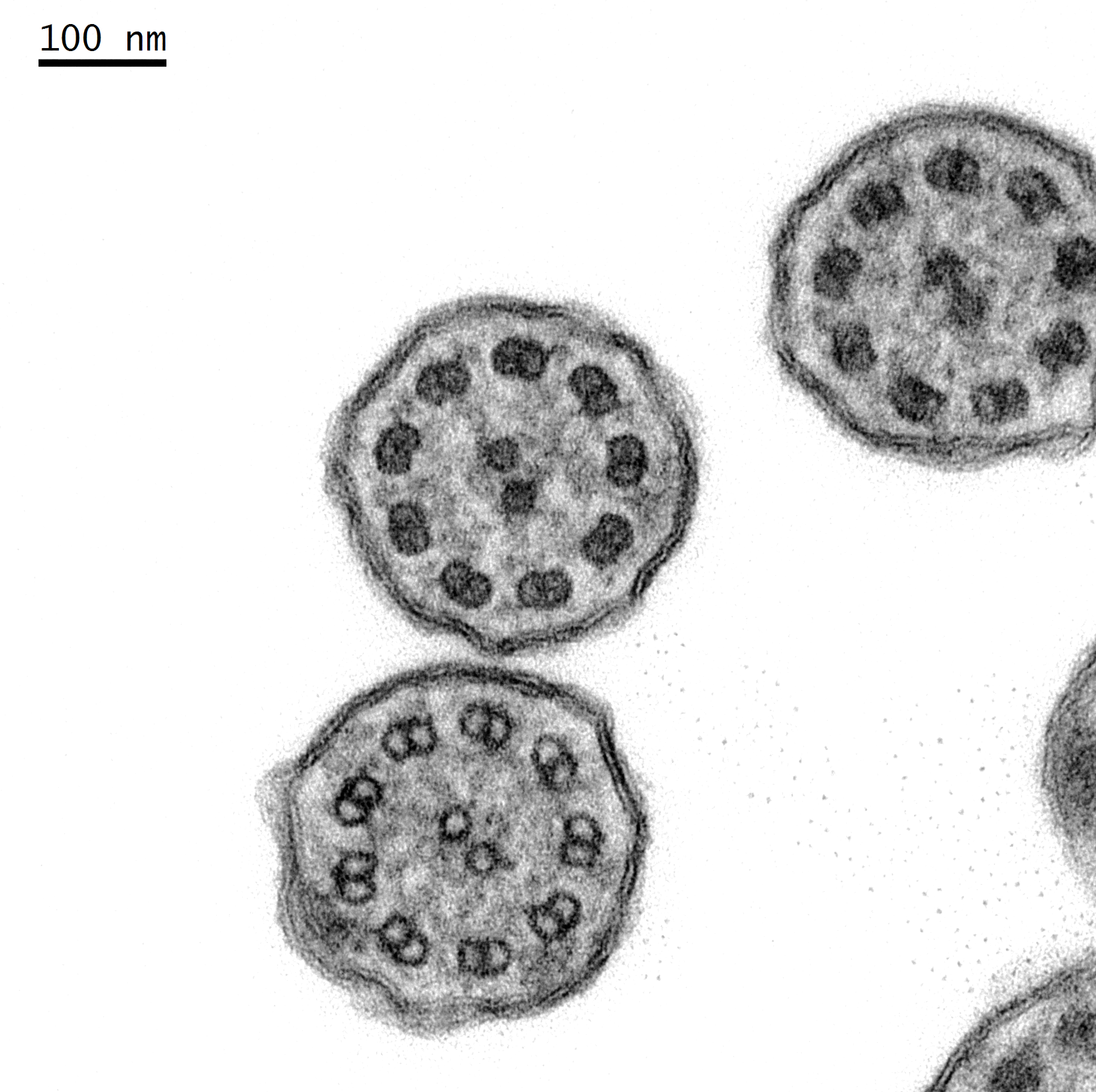


**A**

**B**

**Patient-2**: Absence of both dynein arms (dotted lines exemplified in some doublets) and nexin links (dotted lines exemplified between some doublets). Normal presence of the central pair (circle) and radial spokes (black line exemplified between the central pair and doublet). Doublets have a microtubule A with dynein arms and a microtubule B.


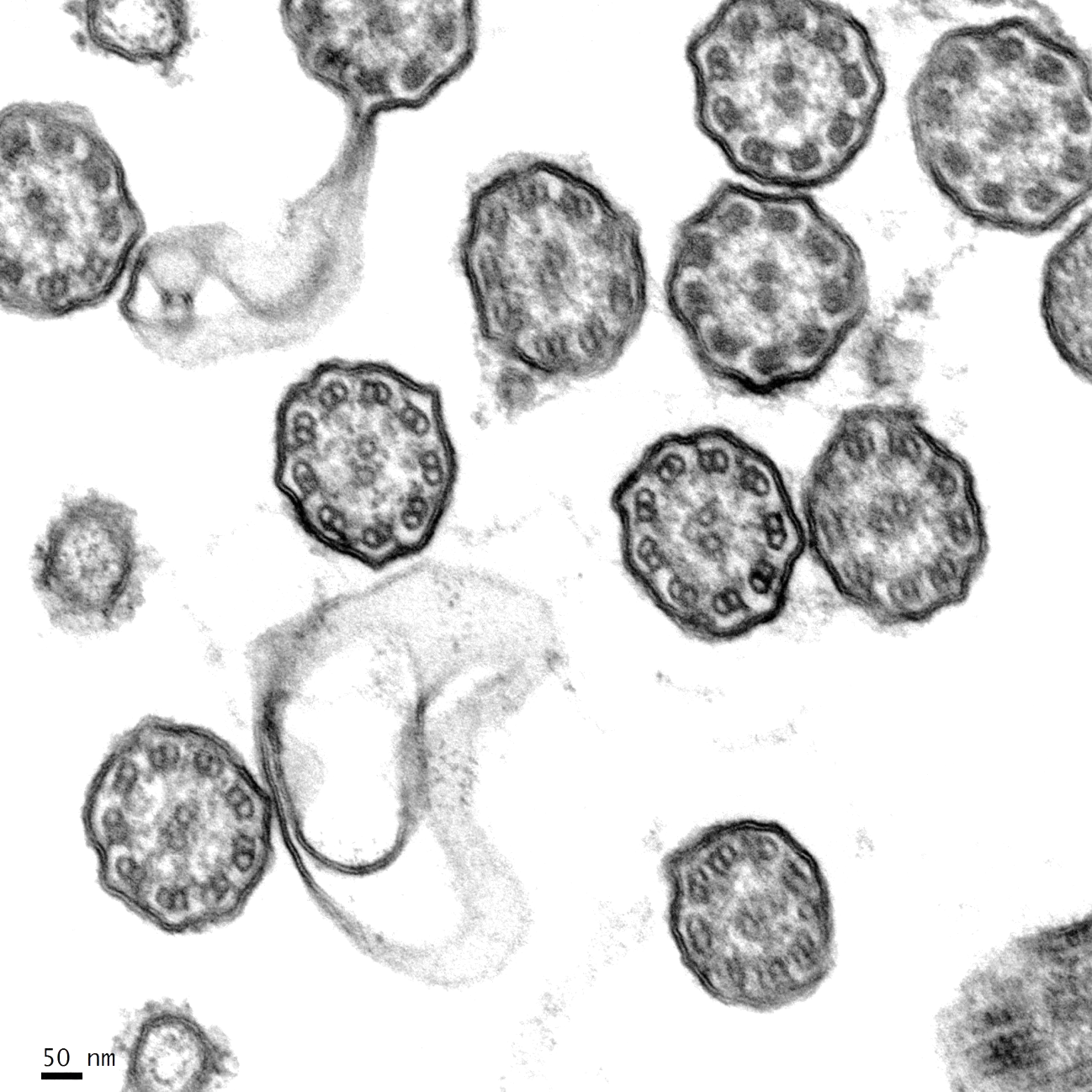


**A**

**B**

**Patient-2**: Absence of both dynein arms (dotted lines exemplified in some doublets) and nexin links (dotted lines exemplified between some doublets). Normal presence of the central pair (circle) and radial spokes (black line exemplified between the central pair and doublet). Doublets have a microtubule A with dynein arms and a microtubule B.
