# Supplementary material for: Clinical and Genetic Analysis of Children with Kartagener Syndrome
Source: Cells. 2019 Aug 15;8(8):900. doi: 10.3390/cells8080900 (PMC6721662; doi:10.3390/cells8080900)
Supplement: Supplementary file 1 [file cells-08-00900-s001.zip › cells-546194-supplementary/Supplementary Table S1.docx]

| Metrics | Patient 1 | Patient 2 | |
| --- | --- | --- | --- |
| Exome-sequencing quality metrics | | |  |
| Total reads (number) | 54 555 040 | 51 474 837 | |
| Aligned reads passed filtering (%) | 99.41 | 99.67 | |
| Mean coverage of target region (x) | 165.7 | 167.6 | |
| Bases with q>20 (number) | 8 824 692 118 | 9 139 618 496 | |
| Uniformity (%) | 88.03 | 91.49 | |
| Captured regions with coverage >20 (%) | 93.64 | 95.63 | |
| Exome variant metrics | | |  |
| Number of homozygous SNVs | 18444 | 19030 | |
| Number of homozygous MNVs | 131 | 148 | |
| Number of homozygous indels | 1122 | 1157 | |
| Number of heterozygous SNVs | 31199 | 30806 | |
| Number of heterozygous MNVs | 306 | 294 | |
| Number of heterozygous indels | 1958 | 1951 | |
| Ti/tv ratio (SNVs) | 2.549 | 2.506 | |
| Novel variants* | 1533 | 1471 | |

**Supplementary Table S1**

Exome-sequencing quality and variant metrics

SNP = single nucleotide polymorphism; SNV = single nucleotide variants; MNV = multiple nucleotide variants; Ti/Tv ratio = the number of transition SNV divided by the number of transversion SNPs. In a WES analysis, a standard Ti/Tv ratio is between 2 to 3. * = novel variants were considered those not listed in dbSNP (eg., no “rs” number attributed).
