# Supplementary material for: Clinical and Genetic Analysis of Children with Kartagener Syndrome
Source: Cells. 2019 Aug 15;8(8):900. doi: 10.3390/cells8080900 (PMC6721662; doi:10.3390/cells8080900)
Supplement: Supplementary file 1 [file cells-08-00900-s001.zip › cells-546194-supplementary/Supplementary Table S2.docx]

**Supplementary Table S2**

List of primers used in both conventional polymerase chain reaction (PCR) and real-time quantitative PCR (qPCR) of this study

| PCR primers | | | |
| --- | --- | --- | --- |
| Gene | **Forward primer (Exon)** | **Reverse primer (Exon)** | **Annealing temperature** |
| *CCDC40*  *NM_017950.3* | AGAAGCTCACCACCCAGTGC (12) | CAACCTGAACACCATGGAAAGG (12) | 58 |
|  | AAACGTTTGCATAAGGAGCCC (17) | CCTGGAAATCTCACCCATTGC (17) | 58 |
| *DNAH5*  *NM_001369.2* | AAATACTAGCAGACCGTCTTGGTCC (28) | GCAGAATCTGTCCCATCTTAGGC (28) | 60 |
|  | ATCTTGTGTGCGTTTCATGCC (36) | GCATCAAGTGACCCAAAACAGC (36) | 60 |
| *DNAH7*  *NM_018897.2* | ACTGTCACCATTTCTACCCAGTGA (44) | AGTTGGCATAATCTTGCCTCCAGT (44) | 58 |
|  | TCAGTGTGGCAATTCTCTCCTTTG (65) | GCTTTCTCTACTCAGCCAGCCA (65) | 58 |
| qPCR primers | | | |
| Gene | **Forward primer** | **Reverse primer** | **Annealing temperature** |
| *DNAH5*  *NM_001369.2* | GCTGGATGACGGTGCAAAACCT | AACCGCTTGGCTTCCTTGGG | 58 |
| *DNAH7*  *NM_018897.2* | GTCTGCCGGTCACTCTTTGA | ATCCAGTCCAATGCCACCAG | 58 |
| *GAPDH*  *NM_001256799.2* | AGGTCGGAGTCAACGGATTT | TGGAATTTGCCATGGGTGGA | 58 |
| *EMC7*  *NM_020154.3* | ATGAGACGGGAAATGGAGCA | CCAGTGTTGCCGTGTTTGTG | 58 |

*CCDC40* = coiled-coil domain containing 40*; DNAH5* = dynein axonemal heavy chain 5; *DNAH7* = dynein axonemal heavy chain 7; *GAPDH* = glyceraldehyde-3-phosphate dehydrogenase; *EMC7* = ER membrane protein complex subunit 7
