# Supplementary material for: Clinical and Genetic Analysis of Children with Kartagener Syndrome
Source: Cells. 2019 Aug 15;8(8):900. doi: 10.3390/cells8080900 (PMC6721662; doi:10.3390/cells8080900)
Supplement: Supplementary file 1 [file cells-08-00900-s001.zip › cells-546194-supplementary/Supplementary Table S3.docx]

**Supplementary Table S3**

Bioinformatic analysis of *DNAH7* variants found in Patient 2

| **Variant** | **SIFT** | **Poly-**  **Phen-2** | **Align GVGD** | **Mutpred2**  **Score** | **MUpro** | **Envision** | | |
| --- | --- | --- | --- | --- | --- | --- | --- | --- |
|  |  |  |  |  |  | **Prediction** | **AA**  **Polarity (WT🡪 MT)** | **Delta**  **PI** |
| c.8209G>A  (p.Gly2737Ser) | 0.02 | 0.60 | C55 | 0.66 | ΔΔG =-0.37 Decrease stability | 0.93 | Hydrophobic 🡪 Polar | 2.39 |
| c.11947C>T  (p.Arg3983Trp) | 0 | 1 | C65 | 0.92 | ΔΔG = -1.01  Decrease stability | 0.74 | Positive Charge🡪 Hydrophobic | -0.45 |

SIFT (<http://sift.jcvi.org/>) scores from 0-0.05 are considered deleterious and >0.05-1.0 are considered tolerated. PolyPhen-2 (<http://genetics.bwh.harvard.edu/pph2/>) scores range from 0-1, with scores < 0.15 considered benign and > 0.15 considered damaging (scores >0.85 being more confidently predicted to be damaging). AlignGVGD (<http://agvgd.hci.utah.edu/>) combines the biophysical characteristics of aminoacids and protein multiple sequence alignments, with scores divided into seven classes (C65, C55, C45, C35, C25, C15, C0), where the higher-class numbers are more predisposed to affect protein function. The MutPred2 (<http://mutpred.mutdb.org/>) is a machine learning-based method that predicts the variant pathogenicity with a prediction score that is the average of the scores from all networks, ranges between 0-1 and the higher scores (>0.6) reflect a higher probability of pathogenicity. MUpro uses support vector machines to predict protein stability changes for single-site mutations (<http://mupro.proteomics.ics.uci.edu/>). Envision (<https://envision.gs.washington.edu/shiny/envision_new/>) combines variant effect measurements from nine large-scale experimental mutagenesis datasets, with damaging variants having scores of less than one.
