# Supplementary material for: Clinical and Genetic Analysis of Children with Kartagener Syndrome
Source: Cells. 2019 Aug 15;8(8):900. doi: 10.3390/cells8080900 (PMC6721662; doi:10.3390/cells8080900)
Supplement: Supplementary file 1 [file cells-08-00900-s001.zip › cells-546194-supplementary/Supplementary Tables S4 and S5.docx]

**Supplementary Tables S4 and S5**

Supplementary Table S4

Compilation of published variants in the *CCDC40* gene

| Coding change | Protein change | Families (number) | Main Ultrastructural anomalies | *Situs inversus* | Method | Ref.* |
| --- | --- | --- | --- | --- | --- | --- |
| c.940–2A>G |  | 1 | IDA defects and Ax disorganisation | ? | Sanger Sequencing | [34] |
| c.961C>T | p.Arg321Ter | 2 |  | ? |  |  |
| c.2440C>T | p.Arg814Ter | 1 |  | ? |  |  |
| c.687delA | p.Pro229Pro fs58Ter | 2 |  | ? |  |  |
| c.2712–1G>T |  | 4 | IDA defects | ? | WES |  |
| c.1415delG | p.Arg472fs3Ter | 1 | IDA defects | ? | Sanger Sequencing |  |
| c.1006C>T | p.Gln336Ter | 1 |  | ? |  |  |
| c.3175C>T | p.Arg1059Ter | 1 |  | ? |  |  |
| c.1464delC | p.Ile488Ilefs19Ter | 1 | IDA defects and Ax disorganisation | ? | Sanger Sequencing | [34,35] |
| c.1345C>T | p.Arg449Ter | 1 |  | Yes |  |  |
| c.344delC | p.Pro115Argfs52Ter | 2 |  | ? |  |  |
| c.3129delC | p.Asp1043Aspfs36Ter | 2 | IDA defects and Ax disorganisation | ? | Sanger Sequencing | [28, 34] |
| c.248delC | p.Ala83ValfsTer84 | 31 | IDA and central pair defects | Yes | WES/ Sanger Sequencing/  Target NGS panel | [34–37] |
| c.1315C>T | p.Gln439Ter | 1 | IDA defects and Ax disorganisation | Yes | Sanger Sequencing | [28] |
| c.1527_1558del | p.Asp510Serfs22Ter | 2 | IDA defects | Yes |  |  |
| c.1971C>T | p.Gln651Ter | 1 |  | Yes |  |  |
| c.2440C>T | p.Arg814Ter | 1 |  | ? |  |  |
| c.1366C>T |  | 1 |  | Yes |  |  |
| c.1810C>T | p.Gln604Ter | 1 |  | Yes |  |  |
| c.2824_2825insTGT | p.Arg942MetinsW | 2 |  | ? |  |  |
| c.3129delC | p.Phe1044Serfs35Ter | 1 |  | Yes |  |  |
| c.778del | p.Ala83Valfs82Ter | 1 |  | ? |  |  |
| c.960C>T | p.Arg321Ter | 1 |  | ? |  |  |
| ivs11-2A>G |  | 1 |  | Yes |  |  |

Axoneme = Ax; Inner dynein arms = IDA; Next-generation sequencing =NGS; WES = Whole exome sequence; ? = information not provided in the original paper. *reference numbers as appear in the main text

**Supplementary Table S4** **(continued)**

Compilation of published variants in the *CCDC40* gene

| Coding change | Protein change | Families (number) | Main Ultrastructural anomalies | *Situs inversus* | Method | Ref.* |
| --- | --- | --- | --- | --- | --- | --- |
| c.574C>T | p.Gln192Ter | 1 | IDA defects | ? | Sanger Sequencing | [35] |
| c.2119G>T | p.Glu707Ter | 1 |  | Yes |  |  |
| c.2712-1G>T |  | 1 |  | ? |  |  |
| c.2591_2592delCAinsACCG | p.Thr864AsnfsX10 | 1 |  | Yes |  |  |
| c.2920C>T | p.Gln974Ter | 1 |  | ? |  |  |
| c.3242_3245dupGGCG | p.Tyr1083AlafsX104 | 1 |  | Yes |  |  |
| c.2609G>A | p. Arg870His | 1 | IDA defects | ? | WES / Sanger Sequencing | [38] |
| c.2527C>T | p.Leu843Phe | 1 | IDA defects and Ax disorganisation | ? | NGS targeted panel | [39] |
| c.580-1G >C |  | 1 |  | ? |  |  |
| c.1259delA | p.Val421Trpfs*2 | 1 | IDA defects and Ax disorganisation | Yes | NGS targeted panel | [40] |
| ex17-20del |  | 1 |  | Yes |  |  |
| c.1416delG | p.Ile473Phefs*2 | 1 | IDA defects | No | WES/ Sanger Sequencing | [36] |
| c.2441G>A | p.Arg814Ter | 1 |  | Yes |  |  |

Axoneme = Ax; Inner dynein arms = IDA; Next-generation sequencing = NGS; WES = Whole exome sequence; ? = information not provided in the original paper. *reference numbers as appear in the main text

**Supplementary Table S5**

Compilation of published variants in the *DNAH5* gene

| Coding change | Protein change | Families (number) | Main Ultrastructural anomalies | *Situs inversus* | Method | Ref.* |
| --- | --- | --- | --- | --- | --- | --- |
| c.8887C>G | p.Gln2949Glu | 1 | DA defects | ? | Sanger Sequencing | [41] |
| c.8396G>C | p.Arg2799Pro | 1 | ODA defects | ? |  |  |
| c.10426C>T | p.Gln3462Ter | 1 | DA defects | ? |  |  |
| c.7429C>T | p.Gln2463Ter | 1 | ODA defects | ? |  |  |
| c.8440_8447 delGAACCAAA | p.2814fsTer1 | 1 | ODA defects | ? |  |  |
| c.10574G>A | p.Arg3539His | 1 | ODA defects | ? |  |  |
| c.5172A>C | p.Lys1710Asn | 2 | DA defects | ? |  |  |
| c.12747G>T | p.Lys4235Asn | 1 | ODA defects | ? |  |  |
| c.4879C>T | p.Gln1613Ter | 1 |  | ? |  |  |
| c.5156+1G>C |  | 1 |  | ? |  |  |
| c.9040C>T | p.Arg3000Ter | 1 |  | ? |  |  |
| c.11625C>A | p.Ser3861Arg | 1 |  | ? |  |  |
| c.5647C>T | p. Arg1883Ter | 1 | ODA defects^1^ | ? | Sanger sequencing | [42] |
| c.670C>T | p. Arg224Ter | 1 |  | ? |  |  |
| c.3876_4053+158del | p.Glu1279-Lys1351del | 1 |  | ? |  |  |
| c.1089+1G>A |  | 1 |  | ? |  |  |
| c.1108A>T | p.Ile370Phe | 2 |  | ? |  |  |
| c.1645A>G | p.Asn549Asp | 2 |  | ? |  |  |
| c.1667A>G | p.Asp556Gly | 2 |  | ? |  |  |
| c.10365G>C | p.Gln3455His | 3 |  | ? |  |  |
| 12397G>T | p.Glu4133Ter | 2 |  | ? |  |  |
| c.5557A>T | p.Lys1853Ter | 1 |  | ? |  |  |
| c.5710-2A>G | p.Cys1904-Lys1909del | 1 |  | ? |  |  |
| c.1619T>C | p.Phe540Leu | 1 |  | ? |  |  |
| c.10615C>T | p.Arg3539Cys | 1 |  | ? |  |  |
| c.8485G>T | p.Val2829Phe | 2 |  | ? |  |  |
| c.8497C>G | p.Arg2833Gly | 2 |  | ? |  |  |
| c.1645A>G | p.Asn549Asp | 1 |  | ? |  |  |
| c.2253C>A | p.Asn751Lys | 4 |  | ? |  |  |
| c.5146C>T | p.Arg1716Trp | 1 |  | ? |  |  |
| c.7888A>T | p.Arg2630Trp | 1 |  | ? |  |  |
| c.5281C>T | p.Arg1761Ter | 2 | ODA defects^1^ | ? | Sanger sequencing | [42,43] |
| c.8029C>T | p.Arg2677Ter | 2 |  | ? |  |  |
| c.7502G>C | p.Arg2501Pro | 2 |  | ? |  |  |
| c.13486C>T | p.Arg4496Ter | 3 |  | ? |  |  |
| c.6791G>A; c.13194_13197del | p.Ser2264Asn; p.Asp4398Glufs*16 | 2 |  | ? |  |  |
| c.4348C>T | p.Gln1450Ter | 3 | ODA defects | ? | Sanger sequencing | [42,44] |
| c.5545G>A | p.Ala1849Thr | 1 |  | ? | Sanger sequencing | [44] |
| c.6988+2T>C | p.Met754Ilefs*5 | 1 |  | ? |  |  |

Axoneme =Ax; Dynein arms = DA; Outer dynein arms = ODA; Next-generation sequencing = NGS; WES = Whole exome sequence; 1 = ODA defects in all patients, which in some cases are also associated with IDA defects but not individually discriminated; ? = information not provided in the original paper. . *reference numbers as appear in the main text.

**Supplementary Table S5 (continued)**

Compilation of published variants in the *DNAH5* gene

| Coding change | Protein change | Families (number) | Main Ultrastructural anomalies | *Situs inversus* | Method | Ref.* |
| --- | --- | --- | --- | --- | --- | --- |
| c.10815delT | p.Pro3606His fs*23 | 9 | ODA defects | ? | Sanger sequencing | [41,43,44] |
| c.6037C>T | p.Arg2013Ter | 1 | ? | ? | Sanger sequencing | [43] |
| c.13458_13459insT | p.Asn4487fs*1 | 4 | ODA defects | ? |  |  |
| c.232C>T | p.Arg78Ter | 1 |  | ? |  |  |
| c.832delG | p.Ala278Arg fsTer27 | 1 |  | ? |  |  |
| c.1627C>T | p.Gln543Ter | 1 |  | ? |  |  |
| c.ivs17+2T>C |  | 1 |  | ? |  |  |
| c.7914_7915insA | p.Arg2639Thr fs*19 | 1 |  | ? |  |  |
| c.3905delT | p.Leu1302Arg fs*19 | 1 |  | ? |  |  |
| c.ivs27+1G>A |  | 1 |  | ? |  |  |
| c.5147G>T | p.Arg1716Leu | 1 |  | ? |  |  |
| c.5482C>T | p.Gln1828Ter | 1 |  | ? |  |  |
| c.1226C>T | p.Trp3409Ser | 1 |  | ? |  |  |
| c.5599_5600insC | p.Leu1867Phe fs*35 | 1 |  | ? |  |  |
| c.ivs76+5G>A |  | 2 |  | ? |  |  |
| c.7039G>A | p.Glu2347Lys | 1 |  | ? |  |  |
| c.8167C>T | p.Gln2723Ter | 1 |  | ? |  |  |
| c.8404C>T | p.Gln2802Ter | 1 |  | ? |  |  |
| c.13426C>T | p.Arg4476Ter | 1 |  | ? |  |  |
| c.8528T>C | p.Phe2843Ser | 1 |  | ? |  |  |
| c.ivs75–2A>T |  | 1 |  | ? |  |  |
| c.9101delG | p.Gly3034Val fs*22 | 2 | ODA shorter | ? | WES / Sanger sequencing | [39,45] |
| c.2261_2262 insT | Met754Ilefs*5 | 1 | ODA defects | ? | WES / Sanger sequencing | [46] |
| c.1121T>C | p.Ile374Thr | 1 |  | ? |  |  |
| c.3139G>A | p.Gly1047Arg | 1 |  | ? |  |  |
| 4361G>A; c.8910_8911  delATinsG | p.Arg1454Gln; p.Phe2971Ser fs*12 | 2 | ODA defect | ? | Sanger sequencing | [47,48] |
| c.11140A>G | p.Ile3714Val | 1 | ODA defects | ? | Target NGS panel | [37] |
| c.638C>A | p.Pro213Gln | 1 |  | ? |  |  |
| c.6710A>G | p.Asn2237Ser | 1 | ODA defects | ? |  |  |

Axoneme = Ax; Dynein arms = DA; Outer dynein arms = ODA; Next-generation sequencing = NGS; WES = Whole exome sequence; ? = information not provided in the original paper. *reference numbers as appear in the main text

**Supplementary Table S5 (continued)**

Compilation of published variants in the *DNAH5* gene

| Coding change | Protein change | Families (number) | Main Ultrastructural anomalies | *Situs inversus* | Method | Ref.* |
| --- | --- | --- | --- | --- | --- | --- |
| c.5563insA | p.1855Asnfs*5 | 1 | ODA defects | ? | Sanger sequencing | [48] |
| c.8440delGAAccAAA | p.2814fs*1 | 1 |  | ? |  |  |
| c.10555G>C | p.Gly3519Arg | 1 |  | ? |  |  |
| c.ivs74-1G>C |  | 1 |  | ? |  |  |
| c.1828C>T | p.Gln610Ter | 1 |  | ? |  |  |
| c.5130insA | p.Arg1711T fs*36 | 1 |  | ? |  |  |
| c.5367delT | p.Asn1790Ile fsX14 | 1 | ODA defects | ? | Target NGS panel | [39] |
| c.9018C>T | p(=)  (splicing disturbed) | 1 |  | Yes |  |  |
| c.7550_7556 delAGCTGCC | p.Glu2517Glyfs*52 | 1 |  | Yes |  |  |
| c.894C>G | p.Asn298Lys | 1 |  | No |  |  |
| c.5563_5564insA | p.Ile1855Asnfs*6 | 4 | ODA defects | ? | Target NGS panel/ Sanger sequencing | [39,43] |
| c.7778C>T | p.Gly2593Glu | 1 | ? | Yes | WES/ Sanger sequencing | [49] |
| c.13729G>A | p(=) | 1 | ? | Yes |  |  |
| c.1090-6A>G |  | 1 | ODA defects | No | WES / Sanger sequencing | [36] |
| c.6230T>C | p.Phe2077Ser | 1 |  | No |  |  |
| c.8498G>A | p.Arg2833His | 1 |  | No |  |  |
| c.10384C>T | p.Gln3462Ter | 1 |  | Yes |  |  |
| c.9427A>T | p.Lys3143Ter | 1 |  | No |  |  |
| c.5983C>T | p.Arg1995Ter | 3 | ODA defects | Yes | WES / Sanger sequencing | [36,39,45] |
| c.10615C>T | p.Arg3539Cys | 2 | ODA defects | No | WES / Sanger sequencing | [36,42] |
| c.6249G>A | p.Met2083Ile | 2 | ODA defects | Yes | WES / Sanger sequencing | [36,46] |

Axoneme = Ax; Dynein arms = DA; Outer dynein arms = ODA; Next-generation sequencing = NGS; WES = Whole exome sequence; ? = information not provided in the original paper. *reference numbers as appear in the main text

**Supplementary References***

28. Becker-Heck, A.; Zohn, I.E.; Okabe, N.; Pollock, A.; Lenhart, K.B.; Sullivan-Brown, J.; McSheene, J.; Loges, N.T.; Olbrich, H.; Haeffner, K.*, et al.* The coiled-coil domain containing protein CCDC40 is essential for motile cilia function and left-right axis formation. *Nat. Genet.* **2011**, *43*, 79-84. doi:<https://doi.org/10.1038/ng.727>.

34. Antony, D.; Becker-Heck, A.; Zariwala, M.A.; Schmidts, M.; Onoufriadis, A.; Forouhan, M.; Wilson, R.; Taylor-Cox, T.; Dewar, A.; Jackson, C.*, et al.* Mutations in CCDC39 and CCDC40 are the Major Cause of Primary Ciliary Dyskinesia with Axonemal Disorganization and Absent Inner Dynein Arms. *Hum. Mutat.* **2013**, *34*, 462-472. doi:<https://doi.org/10.1002/humu.22261>.

35. Blanchon, S.; Legendre, M.; Copin, B.; Duquesnoy, P.; Montantin, G.; Kott, E.; Dastot, F.; Jeanson, L.; Cachanado, M.; Rousseau, A. Delineation of CCDC39/CCDC40 mutation spectrum and associated phenotypes in primary ciliary dyskinesia. *J. Med. Genet.* **2012**, *49*, 410-416. doi:<https://doi.org/10.1136/jmedgenet-2012-100867>.

36. Zariwala, Maimoona A.; Gee, Heon Y.; Kurkowiak, M.; Al-Mutairi, Dalal A.; Leigh, Margaret W.; Hurd, Toby W.; Hjeij, R.; Dell, Sharon D.; Chaki, M.; Dougherty, Gerard W.*, et al.* ZMYND10 Is Mutated in Primary Ciliary Dyskinesia and Interacts with LRRC6. *Am. J. Hum. Genet.* **2013**, *93*, 336-345. doi:<http://dx.doi.org/10.1016/j.ajhg.2013.06.007>.

37. Nakhleh, N.; Francis, R.; Giese, R.A.; Tian, X.; Li, Y.; Zariwala, M.A.; Yagi, H.; Khalifa, O.; Kureshi, S.; Chatterjee, B. High prevalence of respiratory ciliary dysfunction in congenital heart disease patients with heterotaxy. *Circulation* **2012**, *125*, 2232-2242. doi:<https://doi.org/10.1161/CIRCULATIONAHA.111.079780>.

38. Sui, W.; Hou, X.; Che, W.; Ou, M.; Sun, G.; Huang, S.; Liu, F.; Chen, P.; Wei, X.; Dai, Y. CCDC40 mutation as a cause of primary ciliary dyskinesia: a case report and review of literature. *Clin. Respir. J.* **2016**, *10*, 614-621. doi:<https://doi.org/10.1111/crj.12268>.

39. Takeuchi, K.; Kitano, M.; Kiyotoshi, H.; Ikegami, K.; Ogawa, S.; Ikejiri, M.; Nagao, M.; Fujisawa, T.; Nakatani, K. A targeted next-generation sequencing panel reveals novel mutations in Japanese patients with primary ciliary dyskinesia. *Auris Nasus Larynx* **2018**, *45*, 585-591. doi:<https://doi.org/10.1016/j.anl.2017.09.007>.

40. Yang, L.; Banerjee, S.; Cao, J.; Bai, X.; Peng, Z.; Chen, H.; Huang, H.; Han, P.; Feng, S.; Yi, N.*, et al.* Compound Heterozygous Variants in the Coiled-Coil Domain Containing 40 Gene in a Chinese Family with Primary Ciliary Dyskinesia Cause Extreme Phenotypic Diversity in Cilia Ultrastructure. *Frontiers in Genetics* **2018**, *9*, 1-10. doi:<https://doi.org/10.3389/fgene.2018.00023>.

41. Djakow, J.; Svobodová, T.; Hrach, K.; Uhlík, J.; Cinek, O.; Pohunek, P. Effectiveness of sequencing selected exons of DNAH5 and DNAI1 in diagnosis of primary ciliary dyskinesia. *Pediatr. Pulmonol.* **2012**, *47*, 864-875. doi:<https://doi.org/10.1002/ppul.22520>.

42. Failly, M.; Bartoloni, L.; Letourneau, A.; Munoz, A.; Falconnet, E.; Rossier, C.; De Santi, M.M.; Santamaria, F.; Sacco, O.; DeLozier-Blanchet, C.D. Mutations in DNAH5 account for only 15% of a non-preselected cohort of patients with primary ciliary dyskinesia. *J. Med. Genet.* **2009**, *46*, 281-286. doi:<https://doi.org/10.1136/jmg.2008.061176>.

43. Hornef, N.; Olbrich, H.; Horvath, J.; Zariwala, M.a.; Fliegauf, M.; Loges, N.T.; Wildhaber, J.; Noone, P.G.; Kennedy, M.; Antonarakis, S.E.*, et al.* DNAH5 mutations are a common cause of primary ciliary dyskinesia with outer dynein arm defects. *Am. J. Respir. Crit. Care Med.* **2006**, *174*, 120-126. doi:<https://doi.org/10.1164/rccm.200601-084OC>.

44. Ferkol, T.W.; Puffenberger, E.G.; Lie, H.; Helms, C.; Strauss, K.A.; Bowcock, A.; Carson, J.L.; Hazucha, M.; Morton, D.H.; Patel, A.C. Primary ciliary dyskinesia-causing mutations in Amish and Mennonite communities. *J. Pediatr.* **2013**, *163*, 383-387. doi:<http://doi.org/10.1016/j.jpeds.2013.01.061>.

45. Kano, G.; Tsujii, H.; Takeuchi, K.; Nakatani, K.; Ikejiri, M.; Ogawa, S.; Kubo, H.; Nagao, M.; Fujisawa, T. Whole-exome sequencing identification of novel DNAH5 mutations in a young patient with primary ciliary dyskinesia. *Mol. Med. Report.* **2016**, *14*, 5077-5083. doi:<https://doi.org/10.3892/mmr.2016.5871>.

46. Knowles, M.R.; Leigh, M.W.; Ostrowski, L.E.; Huang, L.; Carson, J.L.; Hazucha, M.J.; Yin, W.; Berg, J.S.; Davis, S.D.; Dell, S.D. Exome Sequencing Identifies Mutations in< i> CCDC114</i> as a Cause of Primary Ciliary Dyskinesia. *Am. J. Hum. Genet.* **2013**, *92*, 99-106. doi:<https://doi.org/10.1016/j.ajhg.2012.11.003>.

47. Loges, N.T.; Olbrich, H.; Fenske, L.; Mussaffi, H.; Horvath, J.; Fliegauf, M.; Kuhl, H.; Baktai, G.; Peterffy, E.; Chodhari, R.*, et al.* DNAI2 mutations cause primary ciliary dyskinesia with defects in the outer dynein arm. *Am. J. Hum. Genet.* **2008**, *83*, 547-558. doi:<https://doi.org/10.1016/j.ajhg.2008.10.001>.

48. Olbrich, H.; Häffner, K.; Kispert, A.; Völkel, A.; Volz, A.; Sasmaz, G.; Reinhardt, R.; Hennig, S.; Lehrach, H.; Konietzko, N.*, et al.* Mutations in DNAH5 cause primary ciliary dyskinesia and randomization of left-right asymmetry. *Nat. Genet.* **2002**, *30*, 143-144. doi:<https://doi.org/10.1038/ng817>.

49. Xu, X.; Gong, P.; Wen, J. Clinical and genetic analysis of a family with Kartagener syndrome caused by novel DNAH5 mutations. *J. Assist. Reprod. Genet.* **2017**, *34*, 275-281. doi:<http://doi.org/10.1007/s10815-016-0849-3>.

*reference numbers as appear in the main text
