# Supplementary figures and images for: Clinical and Genetic Analysis of Children with Kartagener Syndrome
Source: Cells. 2019 Aug 15;8(8):900. doi: 10.3390/cells8080900 (PMC6721662; doi:10.3390/cells8080900)

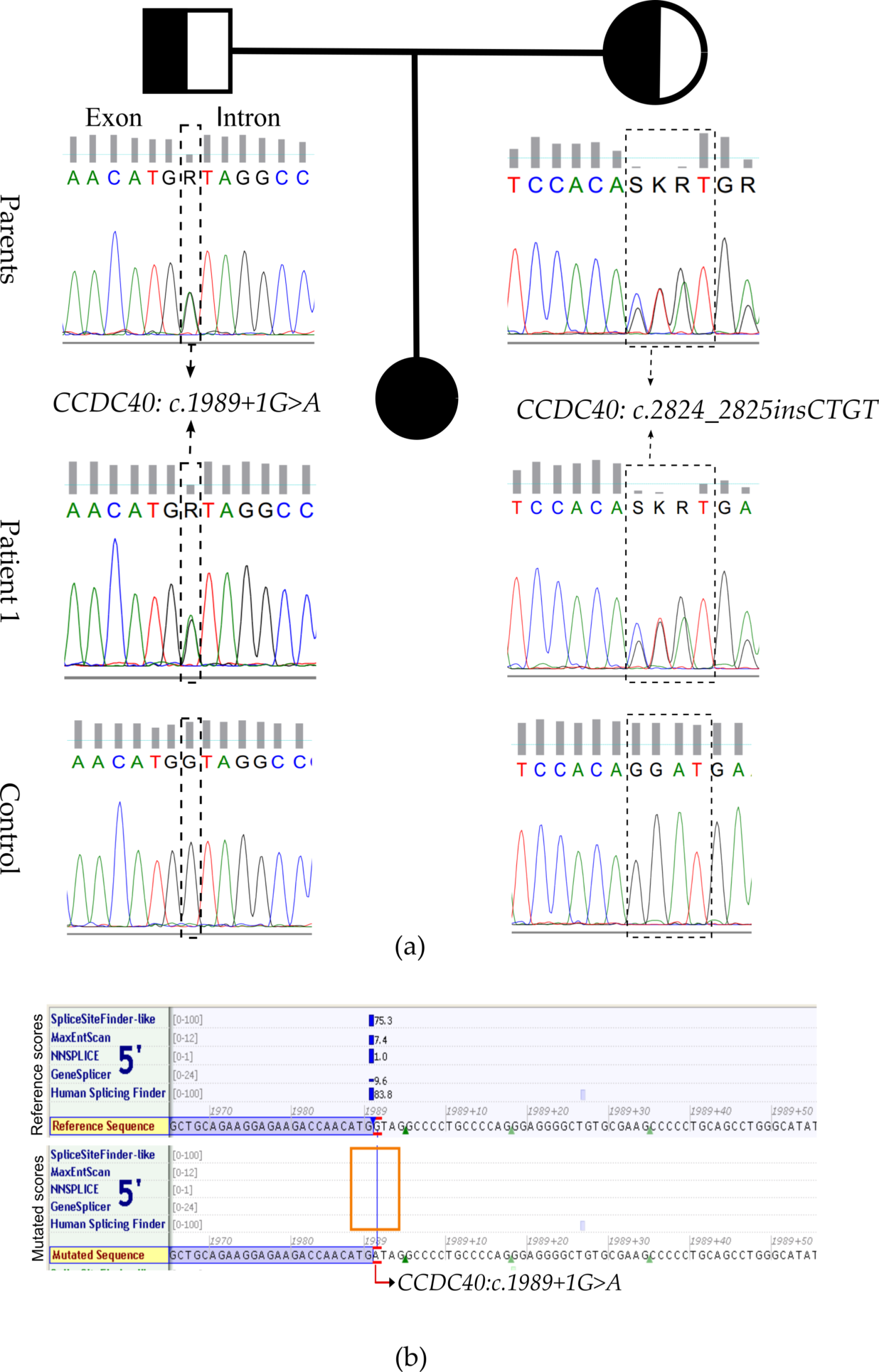

Supplement: Supplementary file 1 [file cells-08-00900-s001.zip › cells-546194-supplementary/Figure-S2.tif]

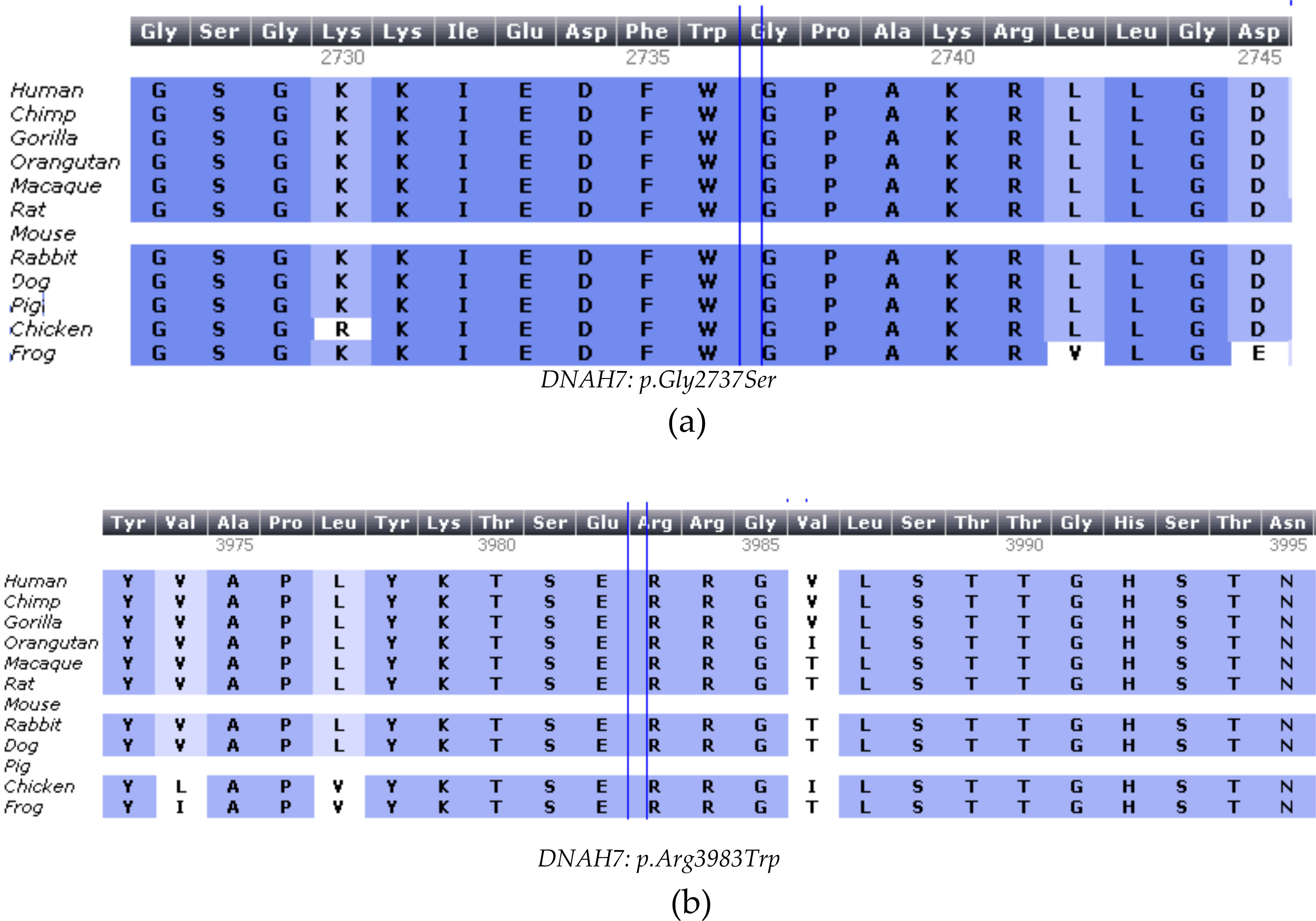

Supplement: Supplementary file 1 [file cells-08-00900-s001.zip › cells-546194-supplementary/Figure-S3.tif]

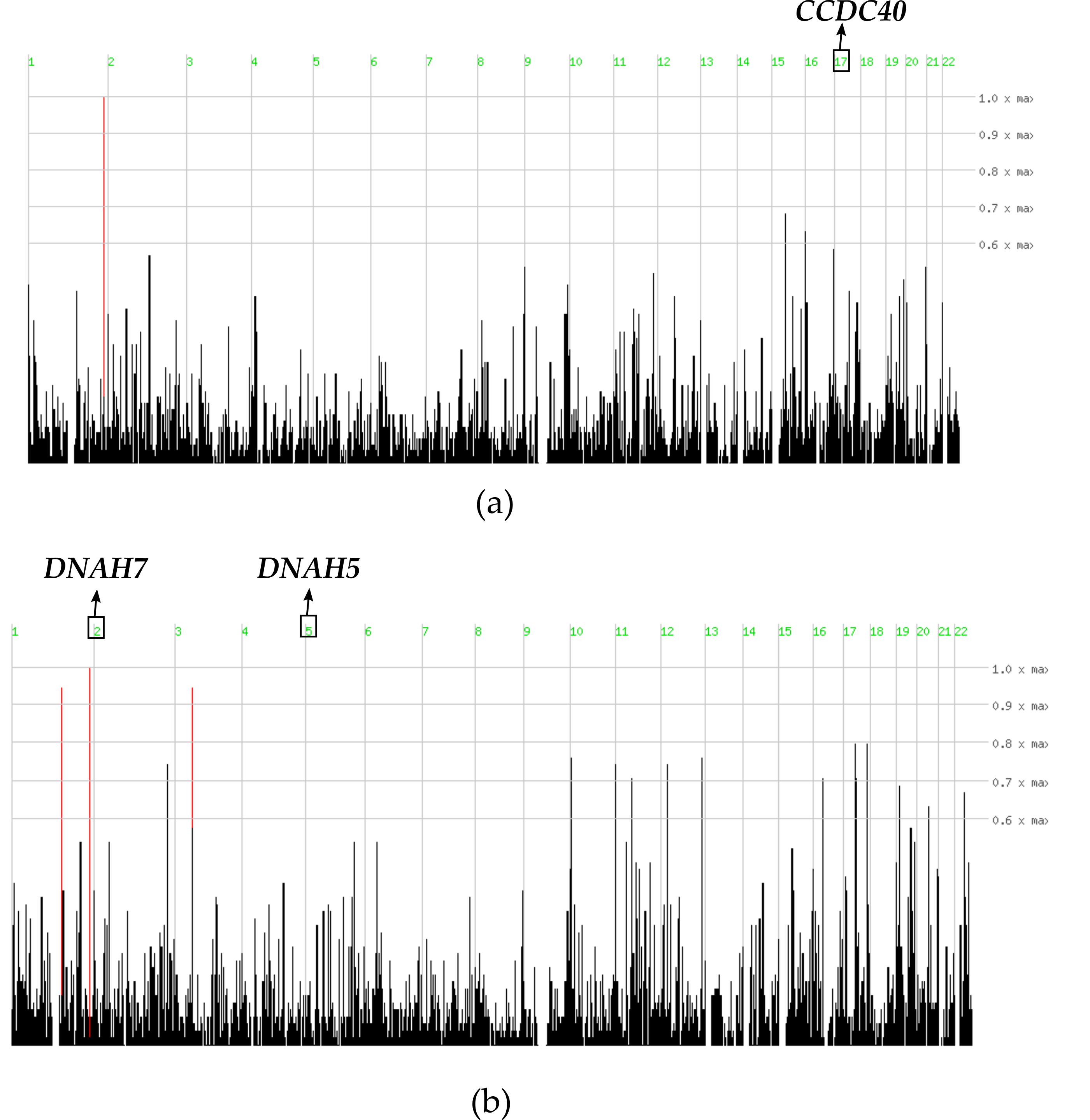

Supplement: Supplementary file 1 [file cells-08-00900-s001.zip › cells-546194-supplementary/Figure-S4.tif]
